# Feasibility of Gamified Mobile Service Aimed at Physical Activation in Young Men: Population-Based Randomized Controlled Study (MOPO)

**DOI:** 10.2196/mhealth.6675

**Published:** 2017-10-10

**Authors:** Anna-Maiju Leinonen, Riitta Pyky, Riikka Ahola, Maarit Kangas, Pekka Siirtola, Tim Luoto, Heidi Enwald, Tiina M Ikäheimo, Juha Röning, Sirkka Keinänen-Kiukaanniemi, Matti Mäntysaari, Raija Korpelainen, Timo Jämsä

**Affiliations:** ^1^ Research Unit of Medical Imaging, Physics and Technology University of Oulu Oulu Finland; ^2^ Infotech Oulu University of Oulu Oulu Finland; ^3^ Oulu Deaconess Institute Department of Sports and Exercise Medicine Oulu Finland; ^4^ Medical Research Center Oulu University Hospital and University of Oulu Oulu Finland; ^5^ Center for Life Course Health Research University of Oulu Oulu Finland; ^6^ Polar Electro Kempele Finland; ^7^ Faculty of Information Technology and Electrical Engineering Biomimetics and Intelligent Systems Group University of Oulu Oulu Finland; ^8^ Department of Cultural Anthropology Faculty of Humanities University of Oulu Oulu Finland; ^9^ Department of Information and Communication Studies Faculty of Humanities University of Oulu Oulu Finland; ^10^ Center for Environmental and Respiratory Health Research University of Oulu Oulu Finland; ^11^ Health Center of Oulu Oulu Finland; ^12^ Center for Military Medicine The Finnish Defence Forces Helsinki Finland; ^13^ Diagnostic Radiology Oulu University Hospital Oulu Finland

**Keywords:** accelerometry, adolescent, behavior change, health, Internet, self-monitoring, wearable

## Abstract

**Background:**

The majority of young people do not meet the recommendations on physical activity for health. New innovative ways to motivate young people to adopt a physically active lifestyle are needed.

**Objective:**

The study aimed to study the feasibility of an automated, gamified, tailored Web-based mobile service aimed at physical and social activation among young men.

**Methods:**

A population-based sample of 496 young men (mean age 17.8 years [standard deviation 0.6]) participated in a 6-month randomized controlled trial (MOPO study). Participants were randomized to an intervention (n=250) and a control group (n=246). The intervention group was given a wrist-worn physical activity monitor (Polar Active) with physical activity feedback and access to a gamified Web-based mobile service, providing fitness guidelines, tailored health information, advice of youth services, social networking, and feedback on physical activity. Through the trial, the physical activity of the men in the control group was measured continuously with an otherwise similar monitor but providing only the time of day and no feedback. The primary outcome was the feasibility of the service based on log data and questionnaires. Among completers, we also analyzed the change in anthropometry and fitness between baseline and 6 months and the change over time in weekly time spent in moderate to vigorous physical activity.

**Results:**

Mobile service users considered the various functionalities related to physical activity important. However, compliance of the service was limited, with 161 (64.4%, 161/250) participants visiting the service, 118 (47.2%, 118/250) logging in more than once, and 41 (16.4%, 41/250) more than 5 times. Baseline sedentary time was higher in those who uploaded physical activity data until the end of the trial (*P*=.02). A total of 187 (74.8%, 187/250) participants in the intervention and 167 (67.9%, 167/246) in the control group participated in the final measurements. There were no differences in the change in anthropometry and fitness from baseline between the groups, whereas waist circumference was reduced in the most inactive men within the intervention group (*P*=.01). Among completers with valid physical activity data (n=167), there was a borderline difference in the change in mean daily time spent in moderate to vigorous physical activity between the groups (11.9 min vs −9.1 min, *P*=.055, linear mixed model). Within the intervention group (n=87), baseline vigorous physical activity was inversely associated with change in moderate to vigorous physical activity during the trial (*R*=−.382, *P*=.01).

**Conclusions:**

The various functionalities related to physical activity of the gamified tailored mobile service were considered important. However, the compliance was limited. Within the current setup, the mobile service had no effect on anthropometry or fitness, except reduced waist circumference in the most inactive men. Among completers with valid physical activity data, the trial had a borderline positive effect on moderate to vigorous physical activity. Further development is needed to improve the feasibility and adherence of an integrated multifunctional service.

**Trial registration:**

Clinicaltrials.gov NCT01376986; http://clinicaltrials.gov/ct2/show/NCT01376986 (Archived by WebCite at http://www.webcitation.org/6tjdmIroA)

## Introduction

The positive effect of physical activity on health [1-3], fitness [4,5], and other lifestyle factors, such as smoking and irregular eating [6], is undeniable. To achieve the health benefits of physical activity, young people under the age of 18 years should accumulate at least 60 min of moderate to vigorous physical activity daily [2]. However, the majority of young people do not meet the global recommendations of physical activity beneficial for their health [7]. Physical activity declines during adolescence even more among boys than girls [8]. Thus, encouraging physical activity among young men is of great importance, and new innovative solutions to motivate young men to adopt a physically active lifestyle are needed [8,9].

Traditional interventions, including face-to-face meetings, consume time and money and are also associated with geographic restrictions [10,11]. On the other hand, interventions delivered through mass media are not able to provide individualized feedback, which has been shown to enhance the effectiveness of physical activity-related interventions [12]. The constantly increasing availability of the Internet and mobile apps offer a new viable way to reach young people easily, even with tailored feedback [13,14]. In addition to tailored and real-time feedback, physical activity profiles, goal setting, social support networking, and online expert consultation have been found to be effective ways to enhance physical activity with smartphone technology [15].

Several previous studies investigating the effect of an Internet-based service or a mobile service for promoting physical activity have shown improved health-related behavior among study participants [16-21]. However, the use of questionnaires to evaluate change in physical activity and the lack of a comparable control group have complicated the investigation of the effect of interventions [15,22]. In addition, intervention studies targeted at young people, especially interventions targeted at boys, are still scarce [12,23].

Nowadays, technology such as wearables enables integrating measured sensor data (eg, physical activity data) directly as parts of mobile services, which further allows delivering automated and tailored feedback messages to the user based on the personal measures. Tailoring of health communication is a means to increase effectiveness of health information by providing more user-centered information [24]. New technology also allows integrating game mechanics to the nongame contexts in a service.

Mobile phone games can be feasible for adolescents to use for promoting physical activity [25]. Gamification means using game design techniques, game principles, and mechanics, such as badges, points, levels, and leaderboards, to improve user engagement, learning, behavior change, and reaching goals [26]. Gamification is increasingly being used for fitness and health-related services to improve user experience and engagement [27], and there are number of health and fitness apps available in the app stores containing at least some components of gamification. Recent reviews have provided an overview of mobile gaming apps to promote daily life physical activity and a demonstration of their acceptability and feasibility among the users [28] and have identified and confirmed the effectiveness of persuasive features in physical activity studies [29]. However, clinical effectiveness and the added value of gaming in changing daily activity behavior have not yet been established. Furthermore, integration of elements of behavioral theory is lacking, which can potentially impact the efficacy of gamification apps to change behavior [30].

In our 3-month MOPO pilot study, we demonstrated that the use of a wearable physical activity monitor providing activity feedback had a short-term positive effect on physical activity behavior among young men [31]. To meet the need for new methods to motivate young people to promote their physical activity, this 6-month trial (ClinicalTrials.gov NCT01376986) evaluated the feasibility of a fully automated, gamified, tailored Web-based mobile service, including physical activity monitoring, in young men. Additionally, we analyzed the effects of the service on anthropometry, fitness, and physical activity among completers. We hypothesized that the mobile service together with continuous physical activity monitoring and feedback is feasible for young men and that the service has a positive effect on anthropometry, fitness, and physical activity.

## Methods

### Study Design and Participants

**Figure 1:**
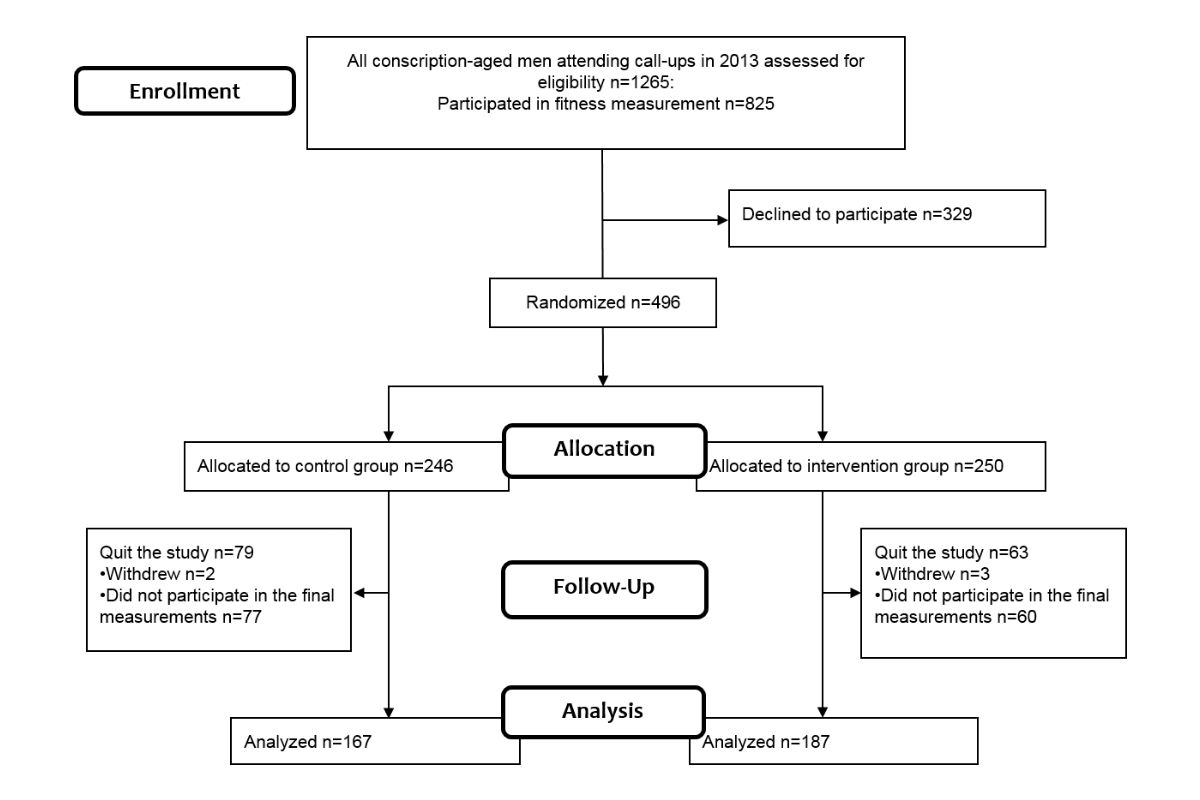
The flow diagram of participants in the 6-month randomized controlled MOPO study.

This 6-month, population-based, parallel randomized controlled trial (MOPO study) was conducted in the city of Oulu, Northern Finland (the number of inhabitants approximately 199,000). The recruitment of the study participants was carried out during the annual military call-ups in September 2013. The military service or civic duty is compulsory for all male citizens in Finland. Finnish Defence Forces organize conscription every year, and all boys turning 18 years participate. During the call-ups in autumn 2013, all conscription-aged men (n=1265) were invited to anthropometry and fitness measurements (Figure 1). All those 825 men (65.21%, 825/1265) who went through the measurements were asked to participate in the 6-month trial. Finally, a total of 496 young men (mean 17.8 years [standard deviation 0.6]) agreed to participate and were randomly allocated (allocation ratio 1:1) to either an intervention group (n=250) or a control group (n=246). Blinded randomization was performed by an assistant who was neither involved in the trial nor in the data collection and analysis. Randomization was conducted based on a list of computer-generated random numbers in blocks of 10. Each participant received sequentially the next random assignment in the list. The reasons for not participating in the trial (n=329) were their lack of interest or laziness (31%); they would not use the wrist-worn clock because they disliked its outlook or already had a wrist watch (23%); they did not feel a need for this type of service; they were already taking care of themselves (9%); and other reasons (17%). In addition, 20% did not give any reason for declining.

The study protocol [32] has been registered to the clinical trials register (NCT01376986, ClinicalTrials.gov). The study participants were provided written and oral information about the procedures of the study, and a written consent was obtained. The study was conducted in accordance to the Declaration of Helsinki. The study was approved by the Local Ethics Committee.

### Intervention

The men in the intervention group had access to an automated, gamified, tailored Web-based mobile physical and social activation service during the trial. The detailed description of the intervention has been published elsewhere [33].

**Figure 2:**
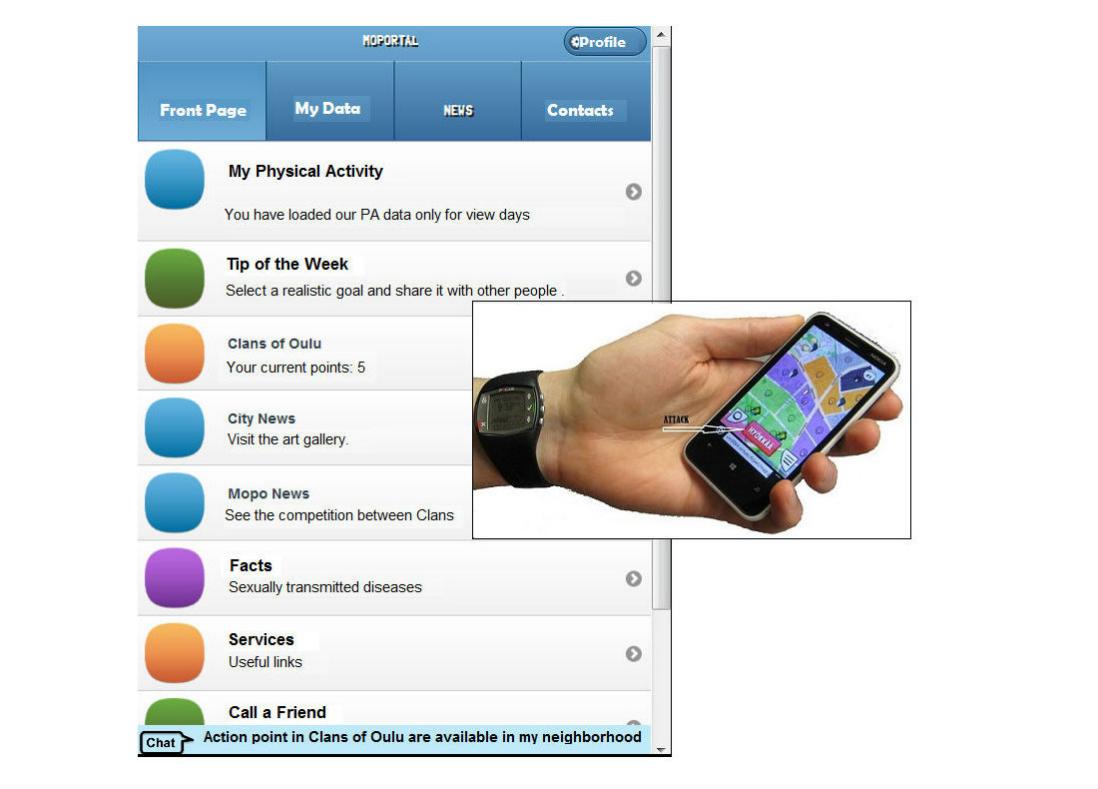
The MOPOrtal service and the Clans of Oulu game.

During the baseline assessments, all participants received a wrist-worn physical activity monitor (Polar Active, Polar Electro). In the intervention group, a personal account for a gamified Web-based mobile service, MOPOrtal (Figure 2), was generated. If necessary, the participants were also provided a mobile phone (n=19) for the duration of the study to be able to use the service. During the first (baseline) week of the trial, the Polar Active monitor was not providing any feedback to the user, and access to the MOPOrtal service was also blocked. After the baseline week, the intervention group was sent a text message (short message service, SMS) instructing how to unlock the monitor screen and log in to MOPOrtal. At the final measurements, the monitors were checked to ascertain whether unlocking had been performed.

Through the trial, the physical activity of the men in the control group was measured continuously with a blinded Polar Active monitor providing only the time of day but no feedback. Otherwise they continued their normal life. The control group had no access to the MOPOrtal service.

All study participants filled in a health and lifestyle questionnaire and went through anthropometry and fitness measurements at baseline in September 2013 and at the end of the trial in March 2014. Those two meetings at baseline and at 6 months were the only face-to-face meetings during the 6-month trial.

### Physical Activity Monitor

Polar Active is a wrist-worn watch-like monitor displaying by default the accumulated daily moderate to vigorous physical activity time and achievement of daily activity target (60 min in this study) as a bar. The time spent on different physical activity levels, steps, and calories for each day are also available for the user through the monitor. The monitor with a 21-day memory is waterproof and includes a uniaxial accelerometer. Polar Active calculates the acceleration signals to metabolic equivalents (MET) with the epoch length of 30 s using sex, age, weight, and height as input. In addition, Polar Active provides time spent in five activity levels using the following thresholds: 1≤ MET<2 (sedentary behaviors), 2≤MET<3.5 (light physical activity), 3.5≤MET<5 (moderate physical activity), 5≤MET<8 (vigorous physical activity), and ≥8 MET (very vigorous physical activity). While assessing energy expenditure, a high correlation has been found between Polar Active and the doubly labeled water technique (*R*=.86), as well as between Polar Active prototype and indirect calorimetry (*R*=.987) [34,35].

The participants in both groups were advised to wear the device on the nondominant wrist at least for all waking hours and to upload their personal activity data to the research database through Polar FlowLink (Polar Electro) at least every 3 weeks. As a reminder to upload the physical activity data, both groups received a text message every 3 weeks. Two movie tickets were raffled once a month as incentives among those participants who uploaded physical activity data.

At least 3 valid days (≥500 min of data) out of 7 were required to be included in the analysis for each week [36]. Mean daily time was calculated for each week for time spent in moderate (3.5-5 MET) and vigorous physical activity (>5 MET) for both groups starting from the next day when the monitor was given. Moderate to vigorous physical activity was defined as the sum of moderate and vigorous physical activity.

### Gamified Mobile Service

The novel gamified mobile service, MOPOrtal (Figure 2), for promoting physical activity and health was set up in the multidisciplinary MOPO study together with the city of Oulu and enterprises of related expertise, including a game studio (LudoCraft Ltd). Additionally, men in the age group of 16 to 20 years from local school classes, voluntary courses, and youth workshops for unemployed young men were involved in designing and testing the service. The underlying idea of the service design was to promote physical and social activity through game-based persuasion, for example, by physically moving within the districts of the city, players could earn points and claim areas for their clan in-game [37]. A sense of affinity was intended to be achieved via a multiplayer cooperation element, as the player belonged to any of the five clans contesting in the game.

The service was running on a Web browser but optimized for mobile use (HTML5), which enabled participants in the intervention group to use it either on a computer or on a mobile device. The activity data, measured using Polar Active, was utilized from the research database to tailor the feedback and information provided by MOPOrtal for each user. The users had also an opportunity to enter daily activity data manually to the service.

The gamified MOPOrtal service has been described in more detail elsewhere [33]. In short, it included (1) automated tailored health information, exercise, and physical activity instructions based on the stage of exercise behavior change; (2) regular automated change check concerning the stage of exercise behavior change; (3) feedback on physical activity and sitting time; (4) *Clans of Oulu* conquering game based on a map and global positioning system (GPS; see below); (5) social networking; and (6) interface to communal youth services and a Web-based helpdesk for technical issues.

**Figure 3:**
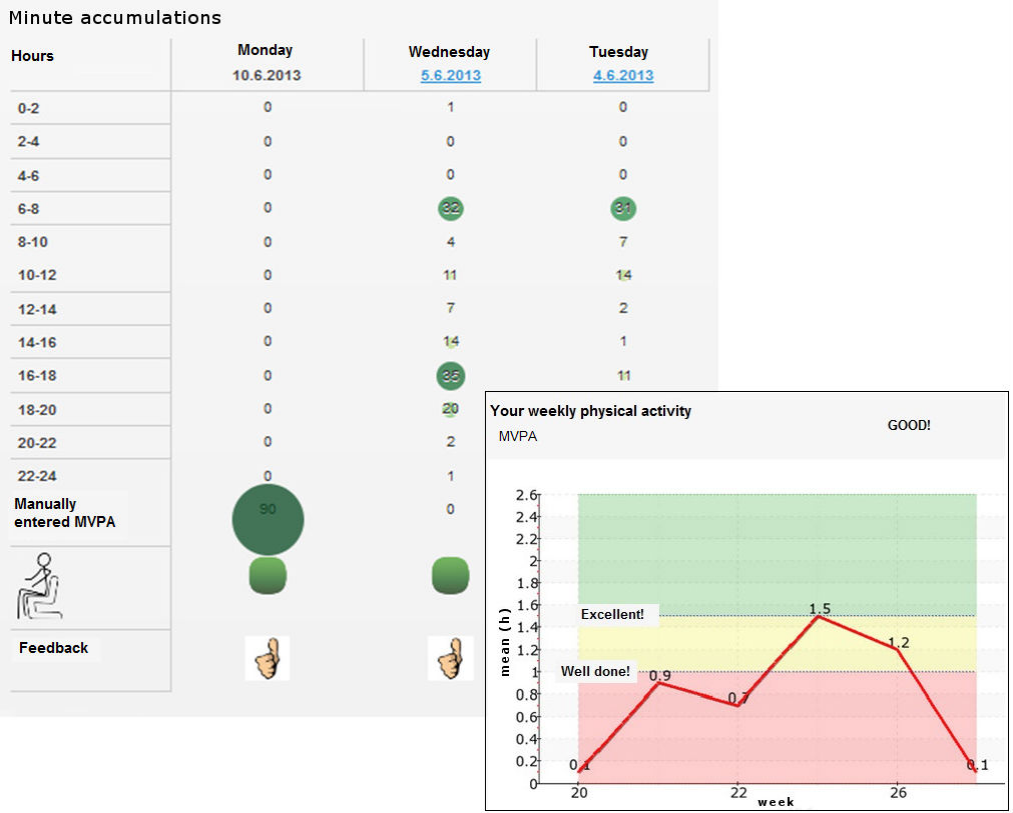
Examples of graphical physical activity and sitting feedback provided by the MOPOrtal service. The line plot represents the weekly feedback (red area—user’s mean average daily moderate to vigorous physical activity (MVPA) is below the global recommendation (60 min/day); yellow and green area—user’s daily moderate to vigorous physical activity level corresponds to the recommendation). The minute accumulations of moderate to vigorous physical activity are given at every 2-hour periods, with the numbers marked as green indicating high accumulation. The thumb gives feedback indicating whether the daily recommendation was fulfilled or not.

Textual and graphical physical activity feedback provided by the service was based on weekly and daily activity metrics (Figure 3). The weekly feedback was given when activity data of at least 3 days out of 7 was available, otherwise the service reminded the user to upload physical activity data from the monitor. The feedback was based on a comparison of user’s activity data to the global recommendation on physical activity for health (60 min/day). Additionally, self-referenced comparison from the previous week was used. Furthermore, the users received positive feedback if their physical activity level was better compared with the weekly average of the whole intervention group (peer-referenced comparison). The overall feedback of daily activity was provided showing a thumb either up, sideways, or down depending on a fulfillment of the global physical activity recommendation and whether the day included over 2 hours of sedentary (sitting) periods or not. In addition, the user was able to see accumulated minutes in different physical activity levels for every 2-hour periods for each day.

The game (*Clans of Oulu* [38]) was based on the location of a person tracked using GPS and played in groups in five different clans using a mobile phone. The basic idea was that by moving physically within the districts of the city of Oulu, players could conquer areas for their own clan. In contrast to traditional games for promoting physical activity, many kinds of activities were rewarded by delivering more points to conquer new areas. For example, uploading of the personal physical activity measures to the research database, fulfillment of daily physical activity recommendation, as well as increment in weekly physical activity and decrement in weekly sedentary time of the player were rewarded. Additionally, by reading facts and health information delivered by the service and inviting friends to join the game, the player received new points to the game. *Clans of Oulu* included, for example, the following game design elements: competition (personal ranks and team ranks), conflict (tasks to be solved and combats with another team), collaboration (working together to reach goals to conquer areas), strategy (points earned based on activity), chance (random new tasks), esthetics (visual appearance for each clan representing different youth cultures), theme (clan game, youth cultures, and conquering), resources (points as resource for concurring areas), time (outdating of points), and scoring or rewards (points for physical activity, completing tasks, and lottery). A more detailed description of the game, its development, and user experiences can be found elsewhere [37].

Gamification was used in MOPOrtal throughout the service, for example, using similar visual appearance as in the *Clans of Oulu* game, and the main actions which occurred in the game (eg, an area occupation from the own clan) were displayed on the service without the need to log in to the game itself.

### Feasibility of the Service

The user-specific information concerning the log-ins to the service and the use of the different service sections were recorded to the research database [39]. In addition, during the final measurements, the participants filled in a questionnaire with questions related to the service and its different components.

### The Transtheoretical Model of Behavior Change

The health information and feedback delivered by the service was tailored based on the transtheoretical model of behavior change (TTM) [40]. The model *is one of the most popular behavior change models utilized in tailored health interventions [41].* Originally, it was developed for smoking cessation, but over the last decades, it has also been used in the background of intervention studies aimed to change exercise and physical activity behavior [42,43]. The model includes five different stages for physical activity adaptation and maintenance: precontemplation, contemplation, preparation, action, and maintenance [40].

Other core constructs of the model are processes of change, decisional balance, and self-efficacy. As individuals proceed to further stages, their confidence in their ability to sustain a target behavior in various situations (self-efficacy) increases, and the advantages of behavior change outweigh the disadvantages leading to decisional balance. The processes of change represent the type of activities that are initiated or experienced by an individual in their attempt to modify affect, behavior, cognitions, or relationships. [40]. Nine processes of change, which include consciousness-raising, dramatic relief, self-reevaluation, and social liberation and the behavioral processes of counterconditioning, helping relationships, reinforcement management, self-liberation, and stimulus control, have received the most empirical support in the context of exercise behavior change [44].

The health information (eg, “Did you know that exercising in nature reduces stress more than exercising in urban environment?”) and individually tailored feedback (eg, “Great! You have sufficient physical activity for your health. If you increase your activity further, your fitness will improve.”) messages delivered automatically by the service were tailored to match the processes of change theorized as most appropriate at each stage [44]. Some modifications were made to the design guide derived from the one designed by Nigg et al. [44]. Furthermore, based on research on feedback perceptions of young men in different stages of exercise behavior change [45,46], message tactics that include self- or peer-referenced comparison, namely normative and ipsative messages, were used only in the more advanced stages (action and maintenance).

In this study, the stage of change was assessed during the first visit to the mobile service based on a modified scale from Cardinal (1995) [47]. The respondents were instructed to choose an alternative that best described their regular exercise behavior and intentions to exercise. Regular exercise was defined according to the Finnish national recommendations for those in the age group of 13 to 18 years as at least 1.5 hours of daily physical activity, of which half should be performed at a vigorous intensity [48]. The answer options were (1) I exercise on a regular basis and have been doing so for longer than 6 months (maintenance), (2) I exercise on a regular basis but I have only begun doing so within the past 6 months (action), (3) I do not exercise, but I have been thinking about starting to exercise within the next month (preparation), (4) I do not exercise, but I have been thinking about starting to exercise within the next 6 months (contemplation), and (5) I do not exercise and do not plan to start exercising in the next 6 months (precontemplation). Additionally, the participants were automatically asked to update their stage of change every 2 months.

### Anthropometry and Fitness

Height and waist circumference (midway between the lowest rib and the iliac crest) were measured with a measuring tape with an accuracy of 0.5 cm. Body composition and weight were assessed by bioelectrical impedance assessment using InBody720 (Biospace Co, Ltd). Bilateral maximal isometric grip strength was measured with a dynamometer while the subject was standing with legs apart and elbow at a 90° angle (SAEHAN Corporation) [49]. The best result of two attempts per hand was recorded. The mean value of both hands was used in the analysis. Polar Fitness Test conducted at rest using FT80 heart rate monitor (Polar Electro) was used to evaluate aerobic fitness. The test assesses maximal oxygen uptake (mL/kg/min) from resting heart rate, heart rate variability, and demographic variables [50]. Polar Fitness Test has been compared with open circuit spirometry for measuring aerobic fitness among adult men with high correlation (.71) and high accuracy (standard error of estimate, SEE=8.5 mL/kg/min) [51]. Additionally, when tested among trained males, the results of Polar Fitness Test were associated highly with laboratory measures (60.2 vs 62.5 mL/kg/min, SEE=7.6 mL/kg/min) [52].

### Outcomes

The primary outcomes were the feasibility of the mobile service. The secondary outcomes were changes from baseline in the anthropometry and fitness and the change in weekly time spent in moderate to vigorous physical activity.

### Statistical Analysis

The results were analyzed with the Statistical Package for the Social Sciences (SPSS) version 19 (IBM Corp) for Windows software. A *P* value below .05 was considered statistically significant. Anthropometry, fitness, and physical activity variables were tested for normality with the Kolmogorov-Smirnov test. The statistical significance of the differences at baseline in continuous variables between the intervention and control groups, between the study participants and nonparticipants, as well as between those young men who based on the log data visited MOPOrtal service at least once and those who did not visit the service at all during the trial were analyzed using the independent samples *t* test. The within-group changes from baseline in the intervention and control groups were analyzed using the paired samples *t* test.

The difference over time in the change in moderate to vigorous physical activity between the intervention and control groups was analyzed using multiple linear mixed model with full maximum likelihood, compound symmetry, and Bonferroni correction. All available personal weekly averages of mean daily time spent in moderate to vigorous physical activity for both study groups were included in the mixed model analyses.

The Pearson correlation coefficient (*R*) was used to evaluate which variables measured at baseline were significantly associated with the main outcome measure in the intervention group. The association between the usage frequency of the service and the occurred change in moderate to vigorous physical activity time was analyzed using the Spearman rank correlation coefficient (ρ).

## Results

### Overview

The baseline characteristics of the study participants were similar between the intervention and control groups (Table 1). In addition, the study participants did not differ in anthropometry and fitness from those conscription-aged men who only took part in the fitness measurements (n=329) but not the trial (data not shown).

In total, 187 (74.8%, 187/250) men in the intervention group and 167 (67.9%, 167/246) in the control group completed the study and attended the final measurements after the 6-month trial (Figure 1). From all study participants, 142 (28.6%, 142/496) did not participate in the final measurements and were excluded from the final analysis.

### Feasibility of the Mobile Service

On the basis of the log data, 161 men (64.4%, 161/250) in the intervention group visited the MOPOrtal service during the trial, 118 (47.2%, 118/250) logged on the service more than once, and 41 (16.4%, 41/250) more than 5 times. In total, 1044 visits were logged (median: 3, range: 1-202). Use rate decreased during the study, being 400 visits during the first month and 69 during the sixth month. A total of 56 participants used the *Clans of Oulu* game in the service. On the basis of the questionnaire, the most common reasons (n=39) for not logging in to the MOPOrtal service at all were (1) not interested or laziness (51%), (2) forgot the service (49%), and (3) technical problems (15%). Among service users, the most common reasons reported for not logging were technical problems or discomfort with the wrist-worn physical activity monitor.

On the basis of the questionnaire (n=94), 90% of MOPOrtal users reported that data related to physical activity (diary and feedback) were important functionalities of the service. Additionally, instructions, test, and goals on physical activity (11%) and general information on health (11%) were also ranked as important functionalities in MOPOrtal. This was also supported by the log data showing that personal data on physical activity was the most used functionality in the service. The service users selected most often sports, movies, or music as their interests. On the other hand, camps, living, well-being, art, theater, or literature were more seldom selected as interests. Mostly, selected personal goals were increasing muscle mass and strength or stamina, whereas the weight control was the most seldom selected. Feedback graphs on daily and weekly physical activity motivated 65% of participants using MOPOrtal service. The reasons why these data did not motivate to move were that these persons were already physically active, they were not interested in physical activity, or they did not need motivation. Additionally, 61% found the feedback messages related to goals to be motivating for physical activity. Tips of the week, including physical activity and wellness messages, were evaluated to be mostly clear, interesting, and reliable.

**Table 1:** Baseline characteristics of the study participants (N=496). Values are mean (standard deviation) unless otherwise specified. N values for the baseline moderate to vigorous physical activity and sedentary time were 87 and 80 for the intervention and control groups, respectively.

Variable	Intervention (n=250)	Control (n=246)
Age, in years	17.9 (0.7)	17.8 (0.6)
Student, n (%)	218 (92.7)	214 (92.2)
Height, cm	177.9 (6.7)	178.1 (6.0)
Weight, kg	73.4 (15.0)	72.9 (14.0)
BMI^a^, kg/m^2^	23.2 (4.5)	23.0 (4.2)
Waist circumference, cm	81.9 (10.9)	81.9 (10.1)
Body fat, %	16.5 (8.5)	16.7 (8.3)
Muscle mass, %	47.1 (4.9)	46.9 (4.9)
Grip strength (mean), kg	45.6 (8.1)	45.6 (7.3)
Estimated maximal aerobic fitness, mL/min/kg	53.6 (7.3)	53.0 (6.8)
Daily moderate to vigorous physical activity^b^ at baseline, min	59.6 (26.2)	61.9 (27.0)
Daily sedentary time at baseline, h	10.4 (2.1)	10.3 (1.9)
At the action or maintenance stage of physical activity^c^ adaption, n (%)	167 (74.2)	152 (68.2)
Current smoker, n (%)	45 (19.6)	48 (21.3)
At least 6 servings of alcohol ≥ once a week, n (%)	43 (20.3)	43 (19.2)

^a^BMI: body mass index.

^b^MVPA: moderate to vigorous physical activity.

^c^PA: physical activity.

Some feedback for further development of MOPOrtal was obtained from the end questionnaire. More visual and clearer user interface (n=6 respondents), more interesting content (n=5), more simple (n=3), or technically more solid and mature solution (n=1) were suggested.

Those men who visited MOPOrtal at least once during the trial had a slightly higher body mass index (BMI) (mean difference of 1.2 kg/m^2^, 95% CI 0.1 kg/m^2^-2.2 kg/m^2^) and body fat percentage (mean difference of 2.2%, 95% CI 0.1%-4.2%) at baseline compared with all other participants of the intervention group. Otherwise, there were no differences in anthropometry and fitness at baseline between these two groups.

By the end of the trial, 178 men in the intervention group (95.2% of those who completed the study) had unlocked the screen of the activity monitor to show daily activity. The total number of valid days of objectively measured activity data was 15,364 (76.4% of all the uploaded data). Data were provided by 138 participants from the intervention group and 138 from the control group. At least 1 valid week was obtained from 120 (48.0%, 120/250) and 110 (44.7%, 110/246) participants in the intervention and control groups, respectively. The average number of valid weeks per person was 10 (median 8) in the intervention and 7 (median 4) in the control group, whereas the average daily usage time of the monitor was 15.1 hours and 15.7 hours in the intervention and control groups, respectively. Valid physical activity data were available from 87 participants in the intervention group and from 80 in the control group at the baseline week, and from 83/70, 56/43, 45/30, and 47/25 participants in the 6-week periods during the follow-up, respectively. Mean daily time occupied in sedentary behavior (1≤ MET< 2) at baseline was significantly higher in those participants who uploaded physical activity data until the end of the trial (n=73) compared with the participants who stopped to deliver data (n=94) during the trial (mean 10.7 hours vs 10.0 hours; *P*=.024, *t* test).

### Effects of Mobile Service on Anthropometry, Fitness, and Physical Activity

Among completers (n=354), there was no statistically significant difference in the change in anthropometry and fitness measurements from baseline between the intervention and control groups. Within the intervention group, the change in the waist circumference differed between TTM-based inactive and active participants (mean change −0.3 cm vs 1.7 cm; *P*=.01, *t* test).

**Figure 4:**
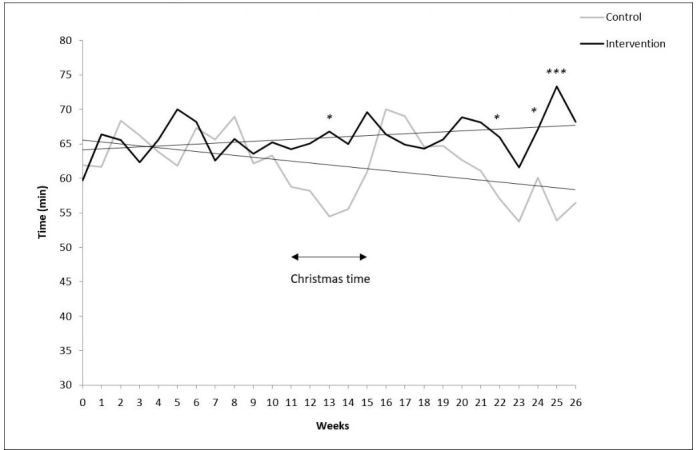
The mean daily time spent in moderate to vigorous physical activity for both study groups as measured by Polar Active during each week of the trial. The average standard deviation was 30.8 min and 24.4 min for the intervention and control groups, respectively. The weeks represent individual weeks from the baseline. Christmas holiday season took place during the study weeks 11 to 15 depending on when the individual started the trial. There was a borderline significant difference between the groups over time (*P*=.055, linear mixed model). MET: metabolic equivalent. * *P*<.05; *** *P*<.001.* *P*<0.05; *** *P*<0.001.

Among completers with valid physical activity data (n=167), there was a borderline difference in the change in mean daily time spent in moderate to vigorous physical activity between the intervention and control groups (11.9 min vs −9.1 min; *P*=.055, linear mixed model; Figure 4). During the last weeks, there was a significant difference between the groups in moderate to vigorous physical activity (*P*<.05 to *P*<.001, *t* test).

The intervention group succeeded to maintain their physical activity level over the Christmas season, whereas physical activity of the control group dropped during the holidays (*P*<.05, *t* test; Figure 4).

Among those men of the intervention group who logged on the service at least once during the trial, the usage frequency of the service was not associated with the change from baseline in the mean daily time spent in moderate to vigorous physical activity (ρ=.045, *P*=.77). Instead, within the intervention group, baseline vigorous physical activity was inversely associated with the change in daily moderate to vigorous physical activity time during the trial (*R*=−.382, *P*=.01).

## Discussion

### Principal Findings

In this 6-month trial, we assessed the feasibility of a fully automated, gamified, tailored Web-based mobile service (MOPOrtal) among young men. Additionally, we evaluated the effects of the service on anthropometry and fitness and objectively measured physical activity. The young men considered the various service functionalities related to physical activity important. The overall compliance was low, varying widely from low to moderate log and data upload frequency. Adherence to upload physical activity data was higher in those participants whose baseline sedentary time was higher. The mobile service had no effect on anthropometry or fitness during the 6-month trial, except reduced waist circumference in the most inactive men. Among the completers with valid physical activity data, there was a positive trend over time in favor of the intervention group in daily time spent in moderate to vigorous physical activity. Low amount of daily vigorous PA at baseline was found to be associated with the increase in moderate to vigorous physical activity during the trial.

The majority of MOPOrtal users perceived the functionalities related to physical activity important, motivating, and related to their personal goals. Both tailoring and gamification were applied to increase compliance by making the service more relevant, engaging, and interesting for the individual. However, the overall compliance was limited. Technical problems, in some degree immature user interface design, and fragmented functionalities were recognized as challenges for the perceived ease of use. To motivate those who are not interested in physical activity but might still benefit from physical activity information and guidance, more persuasive and behavior change–supporting intelligence should be implemented. Compatibility with a variety of sensors and devices would probably increase the usability and feasibility of the service. For successful design of mobile services in future, gamification should be an inseparable and coherent part of the integrity. Additional tailoring, for example, based on physical activity profiling [53], should also be taken into account.

To our knowledge, this is one of the first Web-based physical activity-related intervention studies implemented in a home setting and including young male participants [23]. Unlike many previous studies, this study did not include face-to-face meetings except at baseline and at the end of the trial, allowing the evaluation of the impact based on the Web-based service only [12,22]. The 9-week intervention study conducted among adults with a fully automated Internet-based behavior change system, including continuous-time measurement of physical activity with a wrist-worn accelerometer, achieved consistently a 20 min difference per day in the time spent in moderate physical activity in the intervention group compared with the control group [16]. In addition, a Web-based intervention without any face-to-face meetings in older adults obtained similar results compared with this study, including 11-min increase in time spent in moderate to vigorous physical activity in the intervention group [20]. However, in addition to the difference in the intervention target group, the study duration was only a half, and objective measurement of physical activity was sampled in 7-day intervals at baseline and at 3 months [20].

In our previous pilot study, feedback from a wrist-worn activity monitor had a short-term positive effect on physical activity and sedentary behavior in young men [31]. Here, we observed a trend for a long-term effect in daily moderate to vigorous physical activity time, especially among those with low amount of vigorous physical activity at baseline. However, these data may be biased because of limited sample size, and we cannot distinguish whether the positive effect is a result of the gamified service or the feedback given by the wrist-worn physical activity monitor. The transition from adolescence to adulthood typically includes major life events and is an important phase to interfere with physical activity motivation to prevent negative changes in physical activity behavior and health in future [8]. We implemented different game mechanics in the MOPOrtal service to make it more engaging and attractive. MOPOrtal was a multicomponent service, and the impact of the game that was entered through a portal site cannot be distinguished from the results. However, additional improvements are needed to engage the user to maintain the interest to use the service for a longer time, especially in population-based studies in which the motivation level of the participants may vary. Although the annual military call-ups provide a large truly population-based study sample, this setting may have its challenges in recruiting participants for an eHealth trial, which requires highly motivated and active study participants to succeed [21]. Additionally, the high percentage of young men who declined to participate in the study during the call-ups showed a limited level of motivation among conscription-aged men.

The challenge with low usage of behavioral change services has been revealed also in earlier studies [54]. In several studies the use of an app or a service has dropped after the first month of the study [55]. In a study where a mobile phone app was used together with a face-to-face school-based program in adolescent boys, 20% of participants did not use the app at all [18]. The study using physical activity monitoring and a tailored physical activity coaching website for increasing physical activity reported that only 24% (n=10) of the participants had uploaded physical activity data regularly to the service during the 3-month trial [17]. In our study, physical activity data were provided to the service by 55.2% (138/250) of the participants in the intervention group, and from those, at least 20 weeks of data were obtained only from 21.7% (31/138; data not shown).

As the used behavior change model, that is, TTM, has been originally developed for changing unhealthy behavior [40], it can be discussed whether the model is suitable for the group in which 74.2% (n=167) of the participants are at the action or maintenance stage of physical activity adaptation already at the beginning of the study. The effect of the intervention might have been different if only inactive young men would had been recruited, which was supported by the different change in waist circumference found between inactive and active participants. In addition, at the beginning of the trial, daily objectives were not told, instead, the men from the intervention group achieved the information concerning physical activity recommendation through the MOPOrtal service and physical activity monitor. It is not known whether the information concerning physical activity objectives given by the staff who recruited participants to the trial at baseline would have increased or decreased the compliance of the study.

In this study, the anthropometry at baseline was related to the use of MOPOrtal. Participants who visited MOPOrtal at least once during the trial had a slightly higher BMI and body fat percentage at baseline compared with those who did not use the service at all. In addition, sedentary time at baseline was higher in those participants who uploaded physical activity data until at the end of the trial compared with the participants who stopped to deliver data during the trial. In the study by Tercyak et al, the presence of several behavioral risk factors, such as high BMI and insufficient physical activity, has been positively associated with willingness to use technology for health-promotion purposes among adolescents [56]. In addition, in this 6-month trial, we found out that the anthropometry and fitness at baseline were related to the attendance of the final measurements. Those young men who did not take part in the follow-up measurements had significantly higher body fat as well as lower estimated maximal aerobic fitness and grip strength at baseline compared with all other study participants (data not shown).

There was no change in anthropometry or fitness among the completers in the intervention group except for reduced waist circumference in the most inactive men. This might be because of the low adherence to service use, minor addition in daily moderate to vigorous physical activity time, or the relatively short duration of the trial. In some previous studies, a decrease in body weight and BMI following the use of a mobile phone app has been presented. However, the participants in the successful studies have been adults at increased risk of obesity [55]. A recent study assessing the efficacy of mobile phone technology for the treatment of obesity suggested that some level of counseling is needed in addition to the mobile phone app to improve anthropometry and fitness [57].

On the basis of the physical activity data recorded during this trial, the control group had a drop in moderate to vigorous physical activity during the holiday season in Christmas, followed by the highest peak immediately after Christmas. In contrast, the intervention group maintained their elevated physical activity level during the whole holiday season. In earlier studies, in Western countries, the holiday season in December has been shown to have a negative effect on body fat in college students [58], but the effects of the holiday season on physical activity behavior are not known. However, physical activity behavior shows seasonal differences. After the summer, physical activity usually starts to decline, reaching its lowest level during the winter (January-March) [59,60]. In this study, the corresponding declining trend in time spent in moderate to vigorous physical activity can be seen among the control group, as the trial began in the autumn and ended at the end of the winter season.

### Strengths and Limitations

The main strengths of this study were the large sample size and the population-based randomized controlled design. Another strength was the continuous measurement of physical activity within both study groups, allowing an objective assessment of the change in moderate to vigorous physical activity without any self-reported or user-entered data. The continuous measurement of physical activity also enabled both real-time and Web-based feedback to the intervention group. In addition, the home-based setting (without any face-to-face meetings except at baseline and at 6 months), instead of the more often used school setting, was another strength, allowing for better generalizability of the intervention [23].

One major limitation of this study was the missing physical activity data, the amount of which increased toward the end of the trial. Physical activity data needed to be uploaded to the database at least once every 3 weeks by the study participants, otherwise older data were overwritten by new data. Hence, it may be that some participants in the intervention group considered the feedback given by the PA monitor itself to be enough, and thus, they probably did not see a need for providing their physical activity data to the service. The stored data showed that those participants who did not use the MOPOrtal service, did not upload the physical activity data at all during the trial. More comprehensive self-monitoring with wearables could be obtained with wireless and automatic data transmission. Another limitation was the narrow age range, which limits the generalization of the results. Any long-term follow-up physical activity measurement after the end of the 6-month trial was not conducted in this study, which can also be seen as a limitation.

### Conclusions

The various functionalities related to physical activity of the gamified tailored mobile service were considered important by the young men. However, the compliance to the service was limited. Adherence to upload activity data was higher in those participants whose baseline sedentary time was higher. Within the current setup, the mobile service had no effect on anthropometry or fitness during the 6-month trial, except reduced waist circumference in the most inactive men. Among completers with valid physical activity data, the trial had a borderline positive effect on moderate to vigorous physical activity, especially among those with low amount of vigorous physical activity at baseline. Further development is still needed to improve the feasibility and adherence of an integrated multifunctional service. Mobile services need to be further examined among populations of various ages.

## Abbreviations

BMI: body mass index
GPS: global positioning system
MET: metabolic equivalent
MVPA: moderate-to-vigorous physical activity
PA: physical activity
SEE: standard error of estimate
TTM: transtheoretical model of behavior change

## Appendix

Multimedia Appendix 1 CONSORT-EHEALTH v1.6 checklist.
