# Supplementary material for: Feasibility of Gamified Mobile Service Aimed at Physical Activation in Young Men: Population-Based Randomized Controlled Study (MOPO)
Source: JMIR Mhealth Uhealth. 2017 Oct 10;5(10):e146. doi: 10.2196/mhealth.6675 (PMC5654732; doi:10.2196/mhealth.6675)
Supplement: Multimedia Appendix 1 [file mhealth_v5i10e146_app1.pdf]

CONSORT-EHEALTH (V 1.6.1) - Submission/Publication Form

The CONSORT-EHEALTH checklist is intended for authors of randomized trials evaluating web-based and Internet-based applications/interventions, including mobile interventions, electronic games (incl multiplayer games), social media, certain telehealth applications, and other interactive and/or networked electronic applications. Some of the items (e.g. all subitems under item 5 - description of the intervention) may also be applicable for other study designs.

The goal of the CONSORT EHEALTH checklist and guideline is to be
a) a guide for reporting for authors of RCTs,
b) to form a basis for appraisal of an ehealth trial (in terms of validity)

CONSORT-EHEALTH items/subitems are MANDATORY reporting items for studies published in the Journal of Medical Internet Research and other journals / scientific societies endorsing the checklist.

Items numbered 1., 2., 3., 4a., 4b etc are original CONSORT or CONSORT-NPT (non-pharmacologic treatment) items.
Items with Roman numerals (i., ii, iii, iv etc.) are CONSORT-EHEALTH extensions/clarifications.

As the CONSORT-EHEALTH checklist is still considered in a formative stage, we would ask that you also RATE ON A SCALE OF 1-5 how important/useful you feel each item is FOR THE PURPOSE OF THE CHECKLIST and reporting guideline (optional).

Mandatory reporting items are marked with a red *.
In the textboxes, either copy & paste the relevant sections from your manuscript into this form - please include any quotes from your manuscript in QUOTATION MARKS,
or answer directly by providing additional information not in the manuscript, or elaborating on why the item was not relevant for this study.

YOUR ANSWERS WILL BE PUBLISHED AS A SUPPLEMENTARY FILE TO YOUR PUBLICATION IN JMIR AND ARE CONSIDERED PART OF YOUR PUBLICATION (IF ACCEPTED).
Please fill in these questions diligently. Information will not be copyedited, so please use proper spelling and grammar, use correct capitalization, and avoid abbreviations.

DO NOT FORGET TO SAVE AS PDF _AND_ CLICK THE SUBMIT BUTTON SO YOUR ANSWERS ARE IN OUR DATABASE !!!

Citation Suggestion (if you append the pdf as Appendix we suggest to cite this paper in the caption):
Eysenbach G, CONSORT-EHEALTH Group
CONSORT-EHEALTH: Improving and Standardizing Evaluation Reports of Web-based and Mobile Health Interventions
J Med Internet Res 2011;13(4):e126
URL: http://www.jmir.org/2011/4/e126/
doi: 10.2196/jmir.1923
PMID: 22209829

* Required

Your name *

First Last

Anna-Maiju Leinonen

Primary Affiliation (short), City, Country *

University of Toronto, Toronto, Canada

University of Oulu, Oulı

Your e-mail address *

abc@gmail.com

anna.jauho@oulu.fi

Title of your manuscript *

Provide the (draft) title of your manuscript.

Effect of gamified mobile service on objectively measured physical activity in young men – a population-based randomized controlled MOPO study

Article Preparation Status/Stage *

At which stage in your article preparation are you currently (at the time you fill in this form)

- ○ not submitted yet - in early draft status
- ● not submitted yet - in late draft status, just before submission
- ○ submitted to a journal but not reviewed yet
- ○ submitted to a journal and after receiving initial reviewer comments
- ○ submitted to a journal and accepted, but not published yet
- ○ published
- ○ Other:

Journal *

If you already know where you will submit this paper (or if it is already submitted), please provide the journal name (if it is not JMIR, provide the journal name under "other")

- ○ not submitted yet / unclear where I will submit this
- ● Journal of Medical Internet Research (JMIR)
- ○ Other:

Manuscript tracking number *

If this is a JMIR submission, please provide the manuscript tracking number under "other" (The ms tracking number can be found in the submission acknowledgement email, or when you login as author in JMIR. If the paper is already published in JMIR, then the ms tracking number is the four-digit number at the end of the DOI, to be found at the bottom of each published article in JMIR)

- ● no ms number (yet) / not (yet) submitted to / published in JMIR
- ○ Other:

TITLE AND ABSTRACT

1a) TITLE: Identification as a randomized trial in the title

1a) Does your paper address CONSORT item 1a? *

I.e does the title contain the phrase "Randomized Controlled Trial"? (if not, explain the reason under "other")

- (selected) yes
- Other:

1a-i) Identify the mode of delivery in the title

Identify the mode of delivery. Preferably use "web-based" and/or "mobile" and/or "electronic game" in the title. Avoid ambiguous terms like "online", "virtual", "interactive". Use "Internet-based" only if Intervention includes non-web-based Internet components (e.g. email), use "computer-based" or "electronic" only if offline products are used. Use "virtual" only in the context of "virtual reality" (3-D worlds). Use "online" only in the context of "online support groups". Complement or substitute product names with broader terms for the class of products (such as "mobile" or "smart phone" instead of "iphone"), especially if the application runs on different platforms.

	1	2	3	4	5	
subitem not at all important	○	○	○	○	○	essential

Does your paper address subitem 1a-i? *

Copy and paste relevant sections from manuscript title (include quotes in quotation marks "like this" to indicate direct quotes from your manuscript), or elaborate on this item by providing additional information not in the ms, or briefly explain why the item is not applicable/relevant for your study

"gamified mobile service"

1a-ii) Non-web-based components or important co-interventions in title

Mention non-web-based components or important co-interventions in title, if any (e.g., "with telephone support").

	1	2	3	4	5	
subitem not at all important	○	○	○	○	○	essential

Does your paper address subitem 1a-ii?

Copy and paste relevant sections from manuscript title (include quotes in quotation marks "like this" to indicate direct quotes from your manuscript), or elaborate on this item by providing additional information not in the ms, or briefly explain why the item is not applicable/relevant for your study

Not relevant; Important co-interventions were not used.

1a-iii) Primary condition or target group in the title

Mention primary condition or target group in the title, if any (e.g., “for children with Type I Diabetes”) Example: A Web-based and Mobile Intervention with Telephone Support for Children with Type I Diabetes: Randomized Controlled Trial

	1	2	3	4	5	
subitem not at all important	○	○	○	○	○	essential

Does your paper address subitem 1a-iii? *

Copy and paste relevant sections from manuscript title (include quotes in quotation marks "like this" to indicate direct quotes from your manuscript), or elaborate on this item by providing additional information not in the ms, or briefly explain why the item is not applicable/relevant for your study

"in young men"

1b) ABSTRACT: Structured summary of trial design, methods, results, and conclusions

NPT extension: Description of experimental treatment, comparator, care providers, centers, and blinding status.

1b-i) Key features/functionalities/components of the intervention and comparator in the METHODS section of the ABSTRACT

Mention key features/functionalities/components of the intervention and comparator in the abstract. If possible, also mention theories and principles used for designing the site. Keep in mind the needs of systematic reviewers and indexers by including important synonyms. (Note: Only report in the abstract what the main paper is reporting. If this information is missing from the main body of text, consider adding it)

	1	2	3	4	5	
subitem not at all important	○	○	○	○	○	essential

Does your paper address subitem 1b-i? *

Copy and paste relevant sections from the manuscript abstract (include quotes in quotation marks "like this" to indicate direct quotes from your manuscript), or elaborate on this item by providing additional information not in the ms, or briefly explain why the item is not applicable/relevant for your study

"The intervention group was given a wrist-worn PA monitor (Polar Active) and access to a mobile service, both providing feedback on PA."

1b-ii) Level of human involvement in the METHODS section of the ABSTRACT

Clarify the level of human involvement in the abstract, e.g., use phrases like “fully automated” vs.

“therapist/nurse/care provider/physician-assisted” (mention number and expertise of providers involved, if any). (Note: Only report in the abstract what the main paper is reporting. If this information is missing from the main body of text, consider adding it)

	1	2	3	4	5	
subitem not at all important						essential

Does your paper address subitem 1b-ii?

Copy and paste relevant sections from the manuscript abstract (include quotes in quotation marks "like this" to indicate direct quotes from your manuscript), or elaborate on this item by providing additional information not in the ms, or briefly explain why the item is not applicable/relevant for your study

"a fully automated, gamified, tailored web-based mobile service"

1b-iii) Open vs. closed, web-based (self-assessment) vs. face-to-face assessments in the METHODS section of the ABSTRACT

Mention how participants were recruited (online vs. offline), e.g., from an open access website or from a clinic or a closed online user group (closed usergroup trial), and clarify if this was a purely web-based trial, or there were face-to-face components (as part of the intervention or for assessment). Clearly say if outcomes were self-assessed through questionnaires (as common in web-based trials). Note: In traditional offline trials, an open trial (open-label trial) is a type of clinical trial in which both the researchers and participants know which treatment is being administered. To avoid confusion, use “blinded” or “unblinded” to indicated the level of blinding instead of “open”, as “open” in web-based trials usually refers to “open access” (i.e. participants can self-enrol). (Note: Only report in the abstract what the main paper is reporting. If this information is missing from the main body of text, consider adding it)

	1	2	3	4	5	
subitem not at all important						essential

Does your paper address subitem 1b-iii?

Copy and paste relevant sections from the manuscript abstract (include quotes in quotation marks "like this" to indicate direct quotes from your manuscript), or elaborate on this item by providing additional information not in the ms, or briefly explain why the item is not applicable/relevant for your study

"A population-based sample of 496 conscription-aged (mean 17.8, SD 0.6 years) men participated in a 6-month randomized controlled trial (MOPO study) starting from the annual military call-ups..."
"Main outcome was the change from baseline in objectively measured weekly time spent in moderate-to-vigorous PA (MVPA)... Secondary outcomes included anthropometrics and fitness measurements carried out during the face-to-face meetings at baseline and at 6 months."

1b-iv) RESULTS section in abstract must contain use data

Report number of participants enrolled/assessed in each group, the use/uptake of the intervention (e.g., attrition/adherence metrics, use over time, number of logins etc.), in addition to primary/secondary outcomes. (Note: Only report in the abstract what the main paper is reporting. If this information is missing from the main body of text, consider adding it)

	1	2	3	4	5	
subitem not at all important	○	○	○	○	○	essential

Does your paper address subitem 1b-iv?

Copy and paste relevant sections from the manuscript abstract (include quotes in quotation marks "like this" to indicate direct quotes from your manuscript), or elaborate on this item by providing additional information not in the ms, or briefly explain why the item is not applicable/relevant for your study

"Totally, 187 (74.8%) participants in the intervention group and 167 (67.9%) in the control group completed the study. ...161 (86.1%) participants visited the service, and from them 118 (73.3%) logged in more than once and 41 (25%) more than 5 times." "...mean daily time spent in MVPA increased by 11.9 min...in the intervention group, compared to a mean decrement of 9.1 min...in the control group..." "There was no difference in the change in anthropometrics and fitness measurements from baseline between the two study groups."

1b-v) CONCLUSIONS/DISCUSSION in abstract for negative trials

Conclusions/Discussions in abstract for negative trials: Discuss the primary outcome - if the trial is negative (primary outcome not changed), and the intervention was not used, discuss whether negative results are attributable to lack of uptake and discuss reasons. (Note: Only report in the abstract what the main paper is reporting. If this information is missing from the main body of text, consider adding it)

	1	2	3	4	5	
subitem not at all important	○	○	○	○	○	essential

Does your paper address subitem 1b-v?

Copy and paste relevant sections from the manuscript abstract (include quotes in quotation marks "like this" to indicate direct quotes from your manuscript), or elaborate on this item by providing additional information not in the ms, or briefly explain why the item is not applicable/relevant for your study

"A gamified, tailored mobile service had positive effect on objectively measured MVPA in young men, especially among those with low amount of vigorous PA at baseline. Such service might be used as an early intervention for promoting PA among inactive young men and thereby preventing future adverse health outcomes. "

INTRODUCTION

2a) In INTRODUCTION: Scientific background and explanation of rationale

2a-i) Problem and the type of system/solution

Describe the problem and the type of system/solution that is object of the study: intended as stand-alone intervention vs. incorporated in broader health care program? Intended for a particular patient population? Goals of the intervention, e.g., being more cost-effective to other interventions, replace or complement other solutions? (Note: Details about the intervention are provided in “Methods” under 5)

	1	2	3	4	5	
subitem not at all important	○	○	○	○	○	essential

Does your paper address subitem 2a-i? *

Copy and paste relevant sections from the manuscript (include quotes in quotation marks "like this" to indicate direct quotes from your manuscript), or elaborate on this item by providing additional information not in the ms, or briefly explain why the item is not applicable/relevant for your study

"In order to meet the need for new methods to motivate young people to promote their PA, this 6-month trial evaluated the efficacy of a fully automated, gamified, tailored web-based mobile service including PA monitoring in promoting PA among young men."

2a-ii) Scientific background, rationale: What is known about the (type of) system

Scientific background, rationale: What is known about the (type of) system that is the object of the study (be sure to discuss the use of similar systems for other conditions/diagnoses, if appropiate), motivation for the study, i.e. what are the reasons for and what is the context for this specific study, from which stakeholder viewpoint is the study performed, potential impact of findings [2]. Briefly justify the choice of the comparator.

	1	2	3	4	5	
subitem not at all important	○	○	○	○	○	essential

Does your paper address subitem 2a-ii? *

Copy and paste relevant sections from the manuscript (include quotes in quotation marks "like this" to indicate direct quotes from your manuscript), or elaborate on this item by providing additional information not in the ms, or briefly explain why the item is not applicable/relevant for your study

"Several previous studies investigating the effect of an internet-based service or a mobile service for promoting PA have shown improved health-related behavior among study participants [16-21]. However, use of questionnaires to evaluate change in PA and lack of a comparable control group have complicated the investigation of the effect of intervention [15,22]. In addition, intervention studies targeted at young people, especially interventions for boys, are still scarce [12,23]."

2b) In INTRODUCTION: Specific objectives or hypotheses

Does your paper address CONSORT subitem 2b? *

Copy and paste relevant sections from the manuscript (include quotes in quotation marks "like this" to indicate direct quotes from your manuscript), or elaborate on this item by providing additional information not in the ms, or briefly explain why the item is not applicable/relevant for your study

"We hypothesized that the mobile service together with continuous PA monitoring and feedback increases the PA of young men in long term."

METHODS

3a) Description of trial design (such as parallel, factorial) including allocation ratio

Does your paper address CONSORT subitem 3a? *

Copy and paste relevant sections from the manuscript (include quotes in quotation marks "like this" to indicate direct quotes from your manuscript), or elaborate on this item by providing additional information not in the ms, or briefly explain why the item is not applicable/relevant for your study

"This population-based 6-month parallel randomized controlled trial (MOPO study) was conducted in the city of Oulu, Northern Finland"
"Finally, a total of 496 young (mean 17.8, SD 0.6 years) men agreed to participate and were randomly allocated (allocation ratio 1:1) to either an intervention group (n = 250) or a control group (n = 246)."

3b) Important changes to methods after trial commencement (such as eligibility criteria), with reasons

Does your paper address CONSORT subitem 3b? *

Copy and paste relevant sections from the manuscript (include quotes in quotation marks "like this" to indicate direct quotes from your manuscript), or elaborate on this item by providing additional information not in the ms, or briefly explain why the item is not applicable/relevant for your study

Not relevant; Any changes were not done after the trial commencement.

3b-i) Bug fixes, Downtimes, Content Changes

Bug fixes, Downtimes, Content Changes: ehealth systems are often dynamic systems. A description of changes to methods therefore also includes important changes made on the intervention or comparator during the trial (e.g., major bug fixes or changes in the functionality or content) (5-iii) and other “unexpected events” that may have influenced study design such as staff changes, system failures/downtimes, etc. [2].

1 2 3 4 5

subitem not at all important ○ ○ ○ ○ ○ essential

Does your paper address subitem 3b-i?

Copy and paste relevant sections from the manuscript (include quotes in quotation marks "like this" to indicate direct quotes from your manuscript), or elaborate on this item by providing additional information not in the ms, or briefly explain why the item is not applicable/relevant for your study

Not relevant; Any changes were not done during the trial.

4a) Eligibility criteria for participants

Does your paper address CONSORT subitem 4a? *

Copy and paste relevant sections from the manuscript (include quotes in quotation marks "like this" to indicate direct quotes from your manuscript), or elaborate on this item by providing additional information not in the ms, or briefly explain why the item is not applicable/relevant for your study

"The recruitment of the study participants was carried out during the annual military call-ups in September 2013..." "During the call-ups in autumn 2013 all conscription-aged men (n = 1265) were recruited to voluntary anthropometrics and fitness measurements (Fig. 1). All of those 825 (65.2%) men who went through the measurements were asked to participate to the 6-month trial."

4a-i) Computer / Internet literacy

Computer / Internet literacy is often an implicit “de facto” eligibility criterion - this should be explicitly clarified.

	1	2	3	4	5	
subitem not at all important	○	○	○	○	○	essential

Does your paper address subitem 4a-i?

Copy and paste relevant sections from the manuscript (include quotes in quotation marks "like this" to indicate direct quotes from your manuscript), or elaborate on this item by providing additional information not in the ms, or briefly explain why the item is not applicable/relevant for your study

Not relevant; The target group of the study was young men who are used to use mobile and Internet services.

4a-ii) Open vs. closed, web-based vs. face-to-face assessments:

Open vs. closed, web-based vs. face-to-face assessments: Mention how participants were recruited (online vs. offline), e.g., from an open access website or from a clinic, and clarify if this was a purely

web-based trial, or there were face-to-face components (as part of the intervention or for assessment), i.e., to what degree got the study team to know the participant. In online-only trials, clarify if participants were quasi-anonymous and whether having multiple identities was possible or whether technical or logistical measures (e.g., cookies, email confirmation, phone calls) were used to detect/prevent these.

	1	2	3	4	5	
subitem not at all important	○	○	○	○	○	essential

Does your paper address subitem 4a-ii? *

Copy and paste relevant sections from the manuscript (include quotes in quotation marks "like this" to indicate direct quotes from your manuscript), or elaborate on this item by providing additional information not in the ms, or briefly explain why the item is not applicable/relevant for your study

"During the call-ups in autumn 2013 all conscription-aged men (n = 1265) were recruited to voluntary anthropometrics and fitness measurements (Fig. 1). All of those 825 (65.2%) men who went through the measurements were asked to participate to the 6-month trial."
"...two meetings at baseline and at 6 months were the only face-to-face meetings during the 6-month trial."

4a-iii) Information giving during recruitment

Information given during recruitment. Specify how participants were briefed for recruitment and in the informed consent procedures (e.g., publish the informed consent documentation as appendix, see also item X26), as this information may have an effect on user self-selection, user expectation and may also bias results.

	1	2	3	4	5	
subitem not at all important	○	○	○	○	○	essential

Does your paper address subitem 4a-iii?

Copy and paste relevant sections from the manuscript (include quotes in quotation marks "like this" to indicate direct quotes from your manuscript), or elaborate on this item by providing additional information not in the ms, or briefly explain why the item is not applicable/relevant for your study

"The study participants were provided written and oral information about the procedures of the study, and a written consent was obtained."

4b) Settings and locations where the data were collected

Does your paper address CONSORT subitem 4b? *

Copy and paste relevant sections from the manuscript (include quotes in quotation marks "like this" to indicate direct quotes from your manuscript), or elaborate on this item by providing additional information not in the ms, or briefly explain why the item is not applicable/relevant for your study

"During the call-ups all participants in the intervention group received a wrist-worn PA monitor (Polar Active, Polar Electro, Finland)... During the first (baseline) week of the trial, the Polar Active monitor was not providing any feedback to the user..." "...PA of the men in the control group was measured continuously with a blinded Polar Active monitor... All study participants filled in a health and lifestyle questionnaire and went through anthropometrics and fitness measurements at baseline and at the end of the trial."

4b-i) Report if outcomes were (self-)assessed through online questionnaires

Clearly report if outcomes were (self-)assessed through online questionnaires (as common in web-based trials) or otherwise.

	1	2	3	4	5	
subitem not at all important	○	○	○	○	○	essential

Does your paper address subitem 4b-i? *

Copy and paste relevant sections from the manuscript (include quotes in quotation marks "like this" to indicate direct quotes from your manuscript), or elaborate on this item by providing additional information not in the ms, or briefly explain why the item is not applicable/relevant for your study

Not relevant; Both primary and secondary outcomes were assessed objectively.

4b-ii) Report how institutional affiliations are displayed

Report how institutional affiliations are displayed to potential participants [on ehealth media], as affiliations with prestigious hospitals or universities may affect volunteer rates, use, and reactions with regards to an intervention.(Not a required item – describe only if this may bias results)

	1	2	3	4	5	
subitem not at all important	○	○	○	○	○	essential

Does your paper address subitem 4b-ii?

Copy and paste relevant sections from the manuscript (include quotes in quotation marks "like this" to indicate direct quotes from your manuscript), or elaborate on this item by providing additional information not in the ms, or briefly explain why the item is not applicable/relevant for your study

Not relevant; This multidisciplinary MOPO study was carried in cooperation with the Univesity of Oulu, the city of Oulu, Virpiniemi Sport Institute, Finnish Defence Forces, and enterprises of related expertise.

5) The interventions for each group with sufficient details to allow replication, including how and when they

were actually administered

5-i) Mention names, credential, affiliations of the developers, sponsors, and owners

Mention names, credential, affiliations of the developers, sponsors, and owners [6] (if authors/evaluators are owners or developer of the software, this needs to be declared in a “Conflict of interest” section or mentioned elsewhere in the manuscript).

	1	2	3	4	5	
subitem not at all important	○	○	○	○	○	essential

Does your paper address subitem 5-i?

Copy and paste relevant sections from the manuscript (include quotes in quotation marks "like this" to indicate direct quotes from your manuscript), or elaborate on this item by providing additional information not in the ms, or briefly explain why the item is not applicable/relevant for your study

Not relevant; The service was developed for this particular study.

5-ii) Describe the history/development process

Describe the history/development process of the application and previous formative evaluations (e.g., focus groups, usability testing), as these will have an impact on adoption/use rates and help with interpreting results.

	1	2	3	4	5	
subitem not at all important	○	○	○	○	○	essential

Does your paper address subitem 5-ii?

Copy and paste relevant sections from the manuscript (include quotes in quotation marks "like this" to indicate direct quotes from your manuscript), or elaborate on this item by providing additional information not in the ms, or briefly explain why the item is not applicable/relevant for your study

"The novel mobile service, MOPOrtal (Fig. 2), for promoting PA and health was set up in the multidisciplinary MOPO study together with the city of Oulu and enterprises of related expertise including a game studio (LudoCraft Ltd., Oulu, Finland). Also 16-20 years old men from local school classes, voluntary courses, and youth workshops for unemployed young men were involved in designing and testing the service."

5-iii) Revisions and updating

Revisions and updating. Clearly mention the date and/or version number of the application/intervention (and comparator, if applicable) evaluated, or describe whether the intervention underwent major changes during the evaluation process, or whether the development and/or content was “frozen” during the trial. Describe dynamic components such as news feeds or changing content which may have an impact on the replicability of the intervention (for unexpected events see item 3b).

1 2 3 4 5

subitem not at all important ○ ○ ○ ○ ○ essential

Does your paper address subitem 5-iii?

Copy and paste relevant sections from the manuscript (include quotes in quotation marks "like this" to indicate direct quotes from your manuscript), or elaborate on this item by providing additional information not in the ms, or briefly explain why the item is not applicable/relevant for your study

Not relevant; The service was developed for this particular study.

5-iv) Quality assurance methods

Provide information on quality assurance methods to ensure accuracy and quality of information provided [1], if applicable.

	1	2	3	4	5	
subitem not at all important	○	○	○	○	○	essential

Does your paper address subitem 5-iv?

Copy and paste relevant sections from the manuscript (include quotes in quotation marks "like this" to indicate direct quotes from your manuscript), or elaborate on this item by providing additional information not in the ms, or briefly explain why the item is not applicable/relevant for your study

"While assessing energy expenditure, a high correlation has been found between Polar Active and the doubly labeled water technique (r = 0.86) as well as between Polar Active prototype and indirect calorimetry (r = 0.987) [29,30]"

5-v) Ensure replicability by publishing the source code, and/or providing screenshots/screen-capture video, and/or providing flowcharts of the algorithms used

Ensure replicability by publishing the source code, and/or providing screenshots/screen-capture video, and/or providing flowcharts of the algorithms used. Replicability (i.e., other researchers should in principle be able to replicate the study) is a hallmark of scientific reporting.

	1	2	3	4	5	
subitem not at all important	○	○	○	○	○	essential

Does your paper address subitem 5-v?

Copy and paste relevant sections from the manuscript (include quotes in quotation marks "like this" to indicate direct quotes from your manuscript), or elaborate on this item by providing additional information not in the ms, or briefly explain why the item is not applicable/relevant for your study

Fig. 2: The screenshots from the MOPOrtal service and the Clans of Oulu game." The algorithms are not public.

5-vi) Digital preservation

Digital preservation: Provide the URL of the application, but as the intervention is likely to change or disappear over the course of the years; also make sure the intervention is archived (Internet Archive, webcitation.org, and/or publishing the source code or screenshots/videos alongside the article). As pages behind login screens cannot be archived, consider creating demo pages which are accessible without login.

	1	2	3	4	5	
subitem not at all important	○	○	○	○	○	essential

Does your paper address subitem 5-vi?

Copy and paste relevant sections from the manuscript (include quotes in quotation marks "like this" to indicate direct quotes from your manuscript), or elaborate on this item by providing additional information not in the ms, or briefly explain why the item is not applicable/relevant for your study

Not applicable; The service is not available anymore after the trial.

5-vii) Access

Access: Describe how participants accessed the application, in what setting/context, if they had to pay (or were paid) or not, whether they had to be a member of specific group. If known, describe how participants obtained “access to the platform and Internet” [1]. To ensure access for editors/reviewers /readers, consider to provide a “backdoor” login account or demo mode for reviewers/readers to explore the application (also important for archiving purposes, see vi).

	1	2	3	4	5	
subitem not at all important	○	○	○	○	○	essential

Does your paper address subitem 5-vii? *

Copy and paste relevant sections from the manuscript (include quotes in quotation marks "like this" to indicate direct quotes from your manuscript), or elaborate on this item by providing additional information not in the ms, or briefly explain why the item is not applicable/relevant for your study

"During the call-ups all participants in the intervention group received a wrist-worn PA monitor (Polar Active, Polar Electro, Finland) and a personal account for a gamified web-based mobile service, MOPOrtal (Fig.2)." "During the first (baseline) week of the trial...access to the MOPOrtal service was also blocked. After the baseline week the intervention group was sent a text message instructing how to unlock the monitor screen and log in to MOPOrtal."

5-viii) Mode of delivery, features/functionalities/components of the intervention and comparator, and the theoretical framework

Describe mode of delivery, features/functionalities/components of the intervention and comparator, and the theoretical framework [6] used to design them (instructional strategy [1], behaviour change techniques, persuasive features, etc., see e.g., [7, 8] for terminology). This includes an in-depth description of the content (including where it is coming from and who developed it) [1],” whether [and how] it is tailored to individual circumstances and allows users to track their progress and receive feedback” [6]. This also includes a description of communication delivery channels and – if computer-mediated communication is a component – whether communication was synchronous or asynchronous [6]. It also includes information on presentation strategies [1], including page design principles, average amount of text on pages, presence of hyperlinks to other resources, etc. [1].

	1	2	3	4	5	
subitem not at all important	○	○	○	○	○	essential

Does your paper address subitem 5-viii? *

Copy and paste relevant sections from the manuscript (include quotes in quotation marks "like this" to indicate direct quotes from your manuscript), or elaborate on this item by providing additional information not in the ms, or briefly explain why the item is not applicable/relevant for your study

"The MOPOrtal service included the following: 1) Exercise and PA instructions as well as guidelines for fitness improvement and weight management 2)Feedback on PA based on personal PA measures and objectives... 3) Clans of Oulu game, which is a conquering game based on a location of a person and real-life events. 4) Tailored, automated health information and feedback messages based on the Transtheoretical model of behavior change [31] 5) Information on communal youth services...6) Social-networking possibilities."

5-ix) Describe use parameters

Describe use parameters (e.g., intended “doses” and optimal timing for use). Clarify what instructions or recommendations were given to the user, e.g., regarding timing, frequency, heaviness of use, if any, or was the intervention used ad libitum.

	1	2	3	4	5	
subitem not at all important	○	○	○	○	○	essential

Does your paper address subitem 5-ix?

Copy and paste relevant sections from the manuscript (include quotes in quotation marks "like this" to indicate direct quotes from your manuscript), or elaborate on this item by providing additional information not in the ms, or briefly explain why the item is not applicable/relevant for your study

"The participants in both groups were advised to wear the device on the non-dominating wrist at least for all waking hours and to upload their personal activity data...at least every 3 weeks."
The service was used ad libitum.

5-x) Clarify the level of human involvement

Clarify the level of human involvement (care providers or health professionals, also technical assistance) in the e-intervention or as co-intervention (detail number and expertise of professionals involved, if any, as well as “type of assistance offered, the timing and frequency of the support, how it is initiated, and the medium by which the assistance is delivered”. It may be necessary to distinguish between the level of human involvement required for the trial, and the level of human involvement required for a routine

application outside of a RCT setting (discuss under item 21 – generalizability).

	1	2	3	4	5	
subitem not at all important	○	○	○	○	○	essential

Does your paper address subitem 5-x?

Copy and paste relevant sections from the manuscript (include quotes in quotation marks "like this" to indicate direct quotes from your manuscript), or elaborate on this item by providing additional information not in the ms, or briefly explain why the item is not applicable/relevant for your study

Not relevant; Human involvement was used only to remind the participants to download the PA data every three weeks using a text messages and during the anthropometrics and fitness measurements at baseline and at the end of the trial.

5-xi) Report any prompts/reminders used

Report any prompts/reminders used: Clarify if there were prompts (letters, emails, phone calls, SMS) to use the application, what triggered them, frequency etc. It may be necessary to distinguish between the level of prompts/reminders required for the trial, and the level of prompts/reminders for a routine application outside of a RCT setting (discuss under item 21 – generalizability).

	1	2	3	4	5	
subitem not at all important	○	○	○	○	○	essential

Does your paper address subitem 5-xi? *

Copy and paste relevant sections from the manuscript (include quotes in quotation marks "like this" to indicate direct quotes from your manuscript), or elaborate on this item by providing additional information not in the ms, or briefly explain why the item is not applicable/relevant for your study

"As a reminder to download the PA data, both groups received a text message every three weeks." "The weekly feedback was given when activity data of at least three days out of seven was available; otherwise the service reminded the user to download PA data from the monitor."

5-xii) Describe any co-interventions (incl. training/support)

Describe any co-interventions (incl. training/support): Clearly state any interventions that are provided in addition to the targeted eHealth intervention, as ehealth intervention may not be designed as stand-alone intervention. This includes training sessions and support [1]. It may be necessary to distinguish between the level of training required for the trial, and the level of training for a routine application outside of a RCT setting (discuss under item 21 – generalizability.

	1	2	3	4	5	
subitem not at all important	○	○	○	○	○	essential

Does your paper address subitem 5-xii? *

Copy and paste relevant sections from the manuscript (include quotes in quotation marks "like this" to

indicate direct quotes from your manuscript), or elaborate on this item by providing additional information not in the ms, or briefly explain why the item is not applicable/relevant for your study

Any co-interventions were not used in this study.

6a) Completely defined pre-specified primary and secondary outcome measures, including how and when they were assessed

Does your paper address CONSORT subitem 6a? *

Copy and paste relevant sections from the manuscript (include quotes in quotation marks "like this" to indicate direct quotes from your manuscript), or elaborate on this item by providing additional information not in the ms, or briefly explain why the item is not applicable/relevant for your study

"Main outcome was the change from baseline in weekly time spent in MVPA...The secondary outcomes were the changes from baseline in the anthropometrics and fitness measurements." "During the call-ups all participants in the intervention group received a wrist-worn PA monitor (Polar Active, Polar Electro, Finland).." "...PA of the men in the control group was measured continuously with a blinded Polar Active.." "All study participants...went through anthropometrics and fitness measurements at baseline and at the end of the trial."

6a-i) Online questionnaires: describe if they were validated for online use and apply CHERRIES items to describe how the questionnaires were designed/deployed

If outcomes were obtained through online questionnaires, describe if they were validated for online use and apply CHERRIES items to describe how the questionnaires were designed/deployed [9].

	1	2	3	4	5	
subitem not at all important	○	○	○	○	○	essential

Does your paper address subitem 6a-i?

Copy and paste relevant sections from manuscript text

Not relevant; Online questionnaires were not used during the trial.

6a-ii) Describe whether and how “use” (including intensity of use/dosage) was defined/measured/monitored

Describe whether and how “use” (including intensity of use/dosage) was defined/measured/monitored (logins, logfile analysis, etc.). Use/adoption metrics are important process outcomes that should be reported in any ehealth trial.

	1	2	3	4	5	
subitem not at all important	○	○	○	○	○	essential

Does your paper address subitem 6a-ii?

Copy and paste relevant sections from manuscript text

"The user-specific information concerning the logins to the service and the use of the different service sections were recorded to the research database."

6a-iii) Describe whether, how, and when qualitative feedback from participants was obtained

Describe whether, how, and when qualitative feedback from participants was obtained (e.g., through emails, feedback forms, interviews, focus groups).

	1	2	3	4	5	
subitem not at all important	○	○	○	○	○	essential

Does your paper address subitem 6a-iii?

Copy and paste relevant sections from manuscript text

The results concerning the focus group can be found in another article (Luoto et al. (2014) Gamified persuasion: User experiences of online activation service).

6b) Any changes to trial outcomes after the trial commenced, with reasons

Does your paper address CONSORT subitem 6b? *

Copy and paste relevant sections from the manuscript (include quotes in quotation marks "like this" to indicate direct quotes from your manuscript), or elaborate on this item by providing additional information not in the ms, or briefly explain why the item is not applicable/relevant for your study

Any changes were not occured.

7a) How sample size was determined

NPT: When applicable, details of whether and how the clustering by care provides or centers was addressed

7a-i) Describe whether and how expected attrition was taken into account when calculating the sample size

Describe whether and how expected attrition was taken into account when calculating the sample size.

	1	2	3	4	5	
subitem not at all important	○	○	○	○	○	essential

Does your paper address subitem 7a-i?

Copy and paste relevant sections from manuscript title (include quotes in quotation marks "like this" to indicate direct quotes from your manuscript), or elaborate on this item by providing additional information not in the ms, or briefly explain why the item is not applicable/relevant for your study

See the protocol paper Ahola et al. (2013) Gamified physical activation of young men – a Multidisciplinary Population-Based Randomized Controlled Trial (MOPO study).

7b) When applicable, explanation of any interim analyses and stopping guidelines

Does your paper address CONSORT subitem 7b? *

Copy and paste relevant sections from the manuscript (include quotes in quotation marks "like this" to indicate direct quotes from your manuscript), or elaborate on this item by providing additional information not in the ms, or briefly explain why the item is not applicable/relevant for your study

Not applicable in this study.

8a) Method used to generate the random allocation sequence

NPT: When applicable, how care providers were allocated to each trial group

Does your paper address CONSORT subitem 8a? *

Copy and paste relevant sections from the manuscript (include quotes in quotation marks "like this" to indicate direct quotes from your manuscript), or elaborate on this item by providing additional information not in the ms, or briefly explain why the item is not applicable/relevant for your study

"Randomization was performed after recruitment by an assistant who was neither involved in the trial nor in the data collection and analysis. Randomization was conducted based on a list of computer-generated random numbers in blocks of 10. Each participant received sequentially the next random assignment in the list."

8b) Type of randomisation; details of any restriction (such as blocking and block size)

Does your paper address CONSORT subitem 8b? *

Copy and paste relevant sections from the manuscript (include quotes in quotation marks "like this" to indicate direct quotes from your manuscript), or elaborate on this item by providing additional information not in the ms, or briefly explain why the item is not applicable/relevant for your study

See the section 8a

9) Mechanism used to implement the random allocation sequence (such as sequentially numbered containers), describing any steps taken to conceal the sequence until interventions were assigned

Does your paper address CONSORT subitem 9? *

Copy and paste relevant sections from the manuscript (include quotes in quotation marks "like this" to indicate direct quotes from your manuscript), or elaborate on this item by providing additional information not in the ms, or briefly explain why the item is not applicable/relevant for your study

See the section 8a

10) Who generated the random allocation sequence, who enrolled participants, and who assigned participants to interventions

Does your paper address CONSORT subitem 10? *

Copy and paste relevant sections from the manuscript (include quotes in quotation marks "like this" to indicate direct quotes from your manuscript), or elaborate on this item by providing additional

information not in the ms, or briefly explain why the item is not applicable/relevant for your study

"Randomization was performed after recruitment by an assistant who was neither involved in the trial nor in the data collection and analysis."

11a) If done, who was blinded after assignment to interventions (for example, participants, care providers, those assessing outcomes) and how

NPT: Whether or not administering co-interventions were blinded to group assignment

11a-i) Specify who was blinded, and who wasn't

Specify who was blinded, and who wasn't. Usually, in web-based trials it is not possible to blind the participants [1, 3] (this should be clearly acknowledged), but it may be possible to blind outcome assessors, those doing data analysis or those administering co-interventions (if any).

	1	2	3	4	5	
subitem not at all important	○	○	○	○	○	essential

Does your paper address subitem 11a-i? *

Copy and paste relevant sections from the manuscript (include quotes in quotation marks "like this" to indicate direct quotes from your manuscript), or elaborate on this item by providing additional information not in the ms, or briefly explain why the item is not applicable/relevant for your study

Not applicable; In this study it was not possible to blind the participants after group assignment.

11a-ii) Discuss e.g., whether participants knew which intervention was the "intervention of interest" and which one was the "comparator"

Informed consent procedures (4a-ii) can create biases and certain expectations - discuss e.g., whether participants knew which intervention was the "intervention of interest" and which one was the "comparator".

	1	2	3	4	5	
subitem not at all important	○	○	○	○	○	essential

Does your paper address subitem 11a-ii?

Copy and paste relevant sections from the manuscript (include quotes in quotation marks "like this" to indicate direct quotes from your manuscript), or elaborate on this item by providing additional information not in the ms, or briefly explain why the item is not applicable/relevant for your study

Not relevant; In this study was a true control group which did not receive any intervention actions. "Through the trial, PA of the men in the control group was measured continuously with a blinded Polar Active monitor providing only the time of day. Otherwise the control group continued their normal life."

11b) If relevant, description of the similarity of interventions

(this item is usually not relevant for ehealth trials as it refers to similarity of a placebo or sham intervention to a active medication/intervention)

Does your paper address CONSORT subitem 11b? *

Copy and paste relevant sections from the manuscript (include quotes in quotation marks "like this" to indicate direct quotes from your manuscript), or elaborate on this item by providing additional information not in the ms, or briefly explain why the item is not applicable/relevant for your study

Not relevant in this study.

12a) Statistical methods used to compare groups for primary and secondary outcomes

NPT: When applicable, details of whether and how the clustering by care providers or centers was addressed

Does your paper address CONSORT subitem 12a? *

Copy and paste relevant sections from the manuscript (include quotes in quotation marks "like this" to indicate direct quotes from your manuscript), or elaborate on this item by providing additional information not in the ms, or briefly explain why the item is not applicable/relevant for your study

"Independent samples t-test was used also to analyze the differences in changes from baseline between the two study groups. The within-group changes from baseline in the intervention and control groups were analyzed using the paired samples t-test." "The difference in the change in MVPA from baseline between the intervention and control groups was primarily analyzed using multiple linear regression analyses with GEE [46]."

12a-i) Imputation techniques to deal with attrition / missing values

Imputation techniques to deal with attrition / missing values: Not all participants will use the intervention/comparator as intended and attrition is typically high in ehealth trials. Specify how participants who did not use the application or dropped out from the trial were treated in the statistical analysis (a complete case analysis is strongly discouraged, and simple imputation techniques such as LOCF may also be problematic [4]).

1 2 3 4 5

subitem not at all important ◯ ◯ ◯ ◯ ◯ essential

Does your paper address subitem 12a-i? *

Copy and paste relevant sections from the manuscript (include quotes in quotation marks "like this" to indicate direct quotes from your manuscript), or elaborate on this item by providing additional information not in the ms, or briefly explain why the item is not applicable/relevant for your study

The per protocol analyses were conducted because of the large amount of missing physical activity data: "The difference in the change in MVPA from baseline between the intervention and control groups was primarily analyzed using multiple linear regression analyses with GEE [46]...In addition, the method is not that sensitive with the missing values and it can be used regardless of the type of the dependent variable [47]."

12b) Methods for additional analyses, such as subgroup analyses and adjusted analyses

Does your paper address CONSORT subitem 12b? *

Copy and paste relevant sections from the manuscript (include quotes in quotation marks "like this" to indicate direct quotes from your manuscript), or elaborate on this item by providing additional information not in the ms, or briefly explain why the item is not applicable/relevant for your study

"The Pearson correlation coefficient (R) was used to evaluate which variables measured at baseline were significantly associated with the main outcome measure in the intervention group. The association between the usage frequency of the service and the occurred change in MVPA time was analyzed using the Spearman's rank correlation coefficient (ρ)."

X26) REB/IRB Approval and Ethical Considerations [recommended as subheading under "Methods"] (not a CONSORT item)

X26-i) Comment on ethics committee approval

	1	2	3	4	5	
subitem not at all important	◯	◯	◯	◯	◯	essential

Does your paper address subitem X26-i?

Copy and paste relevant sections from the manuscript (include quotes in quotation marks "like this" to indicate direct quotes from your manuscript), or elaborate on this item by providing additional information not in the ms, or briefly explain why the item is not applicable/relevant for your study

"The study was conducted in accordance to the Declaration of Helsinki. A statement in favor of the study was received from the local Ethics Committee."

x26-ii) Outline informed consent procedures

Outline informed consent procedures e.g., if consent was obtained offline or online (how? Checkbox, etc.?), and what information was provided (see 4a-ii). See [6] for some items to be included in informed consent documents.

	1	2	3	4	5	
subitem not at all important						essential

Does your paper address subitem X26-ii?

Copy and paste relevant sections from the manuscript (include quotes in quotation marks "like this" to indicate direct quotes from your manuscript), or elaborate on this item by providing additional information not in the ms, or briefly explain why the item is not applicable/relevant for your study

"The study participants were provided written and oral information about the procedures of the study, and a written consent was obtained."

X26-iii) Safety and security procedures

Safety and security procedures, incl. privacy considerations, and any steps taken to reduce the likelihood or detection of harm (e.g., education and training, availability of a hotline)

	1	2	3	4	5	
subitem not at all important						essential

Does your paper address subitem X26-iii?

Copy and paste relevant sections from the manuscript (include quotes in quotation marks "like this" to indicate direct quotes from your manuscript), or elaborate on this item by providing additional information not in the ms, or briefly explain why the item is not applicable/relevant for your study

Data were uploaded through a secure Internet connection to the back-up secured research database.

RESULTS

13a) For each group, the numbers of participants who were randomly assigned, received intended treatment, and were analysed for the primary outcome

NPT: The number of care providers or centers performing the intervention in each group and the number of patients treated by each care provider in each center

Does your paper address CONSORT subitem 13a? *

Copy and paste relevant sections from the manuscript (include quotes in quotation marks "like this" to indicate direct quotes from your manuscript), or elaborate on this item by providing additional information not in the ms, or briefly explain why the item is not applicable/relevant for your study

"Totally, 187 (74.8%) men in the intervention group and 167 (67.9%) in the control group completed the study and attended the final measurements after the 6-month trial (Fig. 1). From all study participants, 142 (28.6%) did not participate in the final measurements and were excluded from the final analysis."

13b) For each group, losses and exclusions after randomisation, together with reasons

Does your paper address CONSORT subitem 13b? (NOTE: Preferably, this is shown in a CONSORT flow diagram) *

Copy and paste relevant sections from the manuscript (include quotes in quotation marks "like this" to indicate direct quotes from your manuscript), or elaborate on this item by providing additional information not in the ms, or briefly explain why the item is not applicable/relevant for your study

This is available in the the flow diagram of participants (Fig. 1).

13b-i) Attrition diagram

Strongly recommended: An attrition diagram (e.g., proportion of participants still logging in or using the intervention/comparator in each group plotted over time, similar to a survival curve) or other figures or tables demonstrating usage/dose/engagement.

	1	2	3	4	5	
subitem not at all important	○	○	○	○	○	essential

Does your paper address subitem 13b-i?

Copy and paste relevant sections from the manuscript or cite the figure number if applicable (include quotes in quotation marks "like this" to indicate direct quotes from your manuscript), or elaborate on this item by providing additional information not in the ms, or briefly explain why the item is not applicable/relevant for your study

"...161 men (86.1%) in the intervention group visited the MOPOrtal service during the trial, and from them 118 (73.3%) logged on the service more than once and 41 (25%) more than 5 times. Totally 1044 visits were logged (median 3, range 1-202)."

14a) Dates defining the periods of recruitment and follow-up

Does your paper address CONSORT subitem 14a? *

Copy and paste relevant sections from the manuscript (include quotes in quotation marks "like this" to indicate direct quotes from your manuscript), or elaborate on this item by providing additional information not in the ms, or briefly explain why the item is not applicable/relevant for your study

"All study participants filled in a health and lifestyle questionnaire and went through anthropometrics and fitness measurements at baseline in September 2013 and at the end of the trial in March 2014."

14a-i) Indicate if critical “secular events” fell into the study period

Indicate if critical “secular events” fell into the study period, e.g., significant changes in Internet resources available or “changes in computer hardware or Internet delivery resources”

	1	2	3	4	5	
subitem not at all important	○	○	○	○	○	essential

Does your paper address subitem 14a-i?

Copy and paste relevant sections from the manuscript (include quotes in quotation marks "like this" to indicate direct quotes from your manuscript), or elaborate on this item by providing additional information not in the ms, or briefly explain why the item is not applicable/relevant for your study

Not applicapble; Any critical secular events did not fall into the study period.

14b) Why the trial ended or was stopped (early)

Does your paper address CONSORT subitem 14b? *

Copy and paste relevant sections from the manuscript (include quotes in quotation marks "like this" to indicate direct quotes from your manuscript), or elaborate on this item by providing additional information not in the ms, or briefly explain why the item is not applicable/relevant for your study

Not applicaple in this study.

15) A table showing baseline demographic and clinical characteristics for each group

NPT: When applicable, a description of care providers (case volume, qualification, expertise, etc.) and centers (volume) in each group

Does your paper address CONSORT subitem 15? *

Copy and paste relevant sections from the manuscript (include quotes in quotation marks "like this" to indicate direct quotes from your manuscript), or elaborate on this item by providing additional information not in the ms, or briefly explain why the item is not applicable/relevant for your study

This is shown in Table 1.

15-i) Report demographics associated with digital divide issues

In ehealth trials it is particularly important to report demographics associated with digital divide issues, such as age, education, gender, social-economic status, computer/Internet/ehealth literacy of the participants, if known.

	1	2	3	4	5	
subitem not at all important	○	○	○	○	○	essential

Does your paper address subitem 15-i? *

Copy and paste relevant sections from the manuscript (include quotes in quotation marks "like this" to indicate direct quotes from your manuscript), or elaborate on this item by providing additional information not in the ms, or briefly explain why the item is not applicable/relevant for your study

The mean age and education of the participants are shown in Table 1.

16) For each group, number of participants (denominator) included in each analysis and whether the analysis was by original assigned groups

16-i) Report multiple “denominators” and provide definitions

Report multiple “denominators” and provide definitions: Report N’s (and effect sizes) “across a range of study participation [and use] thresholds” [1], e.g., N exposed, N consented, N used more than x times, N used more than y weeks, N participants “used” the intervention/comparator at specific pre-defined time points of interest (in absolute and relative numbers per group). Always clearly define “use” of the intervention.

	1	2	3	4	5	
subitem not at all important	○	○	○	○	○	essential

Does your paper address subitem 16-i? *

Copy and paste relevant sections from the manuscript (include quotes in quotation marks "like this" to indicate direct quotes from your manuscript), or elaborate on this item by providing additional information not in the ms, or briefly explain why the item is not applicable/relevant for your study

"...161 men (86.1%) in the intervention group visited the MOPOrtal service during the trial, and from them 118 (73.3%) logged on the service more than once and 41 (25%) more than 5 times. Totally 1044 visits were logged (median 3, range 1-202)." See also the flow diagram of participants (Fig.1).

16-ii) Primary analysis should be intent-to-treat

Primary analysis should be intent-to-treat, secondary analyses could include comparing only “users”, with the appropriate caveats that this is no longer a randomized sample (see 18-i).

	1	2	3	4	5	
subitem not at all important	○	○	○	○	○	essential

Does your paper address subitem 16-ii?

Copy and paste relevant sections from the manuscript (include quotes in quotation marks "like this" to indicate direct quotes from your manuscript), or elaborate on this item by providing additional information not in the ms, or briefly explain why the item is not applicable/relevant for your study

The per protocol analyses were conducted because of the large amount of missing physical activity data: "The difference in the change in MVPA from baseline between the intervention and control groups was primarily analyzed using multiple linear regression analyses with GEE [46]...In addition, the method is not that sensitive with the missing values and it can be used regardless of the type of the dependent variable [47]."

17a) For each primary and secondary outcome, results for each group, and the estimated effect size and its precision (such as 95% confidence interval)

Does your paper address CONSORT subitem 17a? *

Copy and paste relevant sections from the manuscript (include quotes in quotation marks "like this" to indicate direct quotes from your manuscript), or elaborate on this item by providing additional information not in the ms, or briefly explain why the item is not applicable/relevant for your study

"There was a statistically significant difference in the change from baseline in mean daily time spent in MVPA between the two study groups (mean difference 21 min; P = .004, t-test) (Fig. 2)...However, no statistically significant intervention effect in MVPA between the two study groups was found over time based on GEE analysis (P = .26)." "There was no statistically significant difference in the change in anthropometrics and fitness measurements from baseline between the intervention and control groups."

17a-i) Presentation of process outcomes such as metrics of use and intensity of use

In addition to primary/secondary (clinical) outcomes, the presentation of process outcomes such as metrics of use and intensity of use (dose, exposure) and their operational definitions is critical. This does not only refer to metrics of attrition (13-b) (often a binary variable), but also to more continuous exposure metrics such as “average session length”. These must be accompanied by a technical description how a metric like a “session” is defined (e.g., timeout after idle time) [1] (report under item 6a).

	1	2	3	4	5	
subitem not at all important	○	○	○	○	○	essential

Does your paper address subitem 17a-i?

Copy and paste relevant sections from the manuscript (include quotes in quotation marks "like this" to indicate direct quotes from your manuscript), or elaborate on this item by providing additional information not in the ms, or briefly explain why the item is not applicable/relevant for your study

"...161 men (86.1%) in the intervention group visited the MOPOrtal service during the trial, and from them 118 (73.3%) logged on the service more than once and 41 (25%) more than 5 times. Totally 1044 visits were logged (median 3, range 1-202). According to log data, the participants were particularly interested in the sections involved in PA, i.e., tracking their own PA and PA feedback."

17b) For binary outcomes, presentation of both absolute and relative effect sizes is recommended

Does your paper address CONSORT subitem 17b? *

Copy and paste relevant sections from the manuscript (include quotes in quotation marks "like this" to indicate direct quotes from your manuscript), or elaborate on this item by providing additional information not in the ms, or briefly explain why the item is not applicable/relevant for your study

Not applicable.

18) Results of any other analyses performed, including subgroup analyses and adjusted analyses, distinguishing pre-specified from exploratory

Does your paper address CONSORT subitem 18? *

Copy and paste relevant sections from the manuscript (include quotes in quotation marks "like this" to indicate direct quotes from your manuscript), or elaborate on this item by providing additional information not in the ms, or briefly explain why the item is not applicable/relevant for your study

"Those men who visited MOPOrtal at least once during the trial had a slightly higher body mass index (mean difference of 1.2 kg/m2, 95% CI: 0.1 kg/m2 to 2.2 kg/m2) and body fat percentage (mean difference of 2.2%, 95% CI: 0.1% to 4.2%) at baseline..." "...within the intervention group, baseline vigorous PA was negatively correlated with the change in daily MVPA time during the trial (R = -0.382, P = .01)."

18-i) Subgroup analysis of comparing only users

A subgroup analysis of comparing only users is not uncommon in ehealth trials, but if done, it must be stressed that this is a self-selected sample and no longer an unbiased sample from a randomized trial (see 16-iii).

	1	2	3	4	5	
subitem not at all important	○	○	○	○	○	essential

Does your paper address subitem 18-i?

Copy and paste relevant sections from the manuscript (include quotes in quotation marks "like this" to indicate direct quotes from your manuscript), or elaborate on this item by providing additional information not in the ms, or briefly explain why the item is not applicable/relevant for your study

"Among those intervention group men who logged on the service at least once during the trial the usage frequency of the service was not associated with the change from baseline in mean daily time spent in MVPA (ρ = 0.045, P = .77)."

19) All important harms or unintended effects in each group

(for specific guidance see CONSORT for harms)

Does your paper address CONSORT subitem 19? *

Copy and paste relevant sections from the manuscript (include quotes in quotation marks "like this" to indicate direct quotes from your manuscript), or elaborate on this item by providing additional information not in the ms, or briefly explain why the item is not applicable/relevant for your study

No harms or side effects were noticed in this study.

19-i) Include privacy breaches, technical problems

Include privacy breaches, technical problems. This does not only include physical “harm” to participants,

but also incidents such as perceived or real privacy breaches [1], technical problems, and other unexpected/unintended incidents. “Unintended effects” also includes unintended positive effects [2].

	1	2	3	4	5	
subitem not at all important	○	○	○	○	○	essential

Does your paper address subitem 19-i?

Copy and paste relevant sections from the manuscript (include quotes in quotation marks "like this" to indicate direct quotes from your manuscript), or elaborate on this item by providing additional information not in the ms, or briefly explain why the item is not applicable/relevant for your study

Not relevant in this study.

19-ii) Include qualitative feedback from participants or observations from staff/researchers

Include qualitative feedback from participants or observations from staff/researchers, if available, on strengths and shortcomings of the application, especially if they point to unintended/unexpected effects or uses. This includes (if available) reasons for why people did or did not use the application as intended by the developers.

	1	2	3	4	5	
subitem not at all important	○	○	○	○	○	essential

Does your paper address subitem 19-ii?

Copy and paste relevant sections from the manuscript (include quotes in quotation marks "like this" to indicate direct quotes from your manuscript), or elaborate on this item by providing additional information not in the ms, or briefly explain why the item is not applicable/relevant for your study

The results concerning the focus group and qualitative feedback from the participants can be found in another article (Luoto et al. (2014) Gamified persuasion: User experiences of online activation service).

DISCUSSION

22) Interpretation consistent with results, balancing benefits and harms, and considering other relevant evidence

NPT: In addition, take into account the choice of the comparator, lack of or partial blinding, and unequal expertise of care providers or centers in each group

22-i) Restate study questions and summarize the answers suggested by the data, starting with

primary outcomes and process outcomes (use)

Restate study questions and summarize the answers suggested by the data, starting with primary outcomes and process outcomes (use).

	1	2	3	4	5	
subitem not at all important	○	○	○	○	○	essential

Does your paper address subitem 22-i? *

Copy and paste relevant sections from the manuscript (include quotes in quotation marks "like this" to indicate direct quotes from your manuscript), or elaborate on this item by providing additional information not in the ms, or briefly explain why the item is not applicable/relevant for your study

"...we assessed the impact of a fully automated, gamified, tailored web-based mobile service (MOPOrtal) on objectively measured physical activity among young men. ...mean daily time spent in moderate-to-vigorous PA increased in the intervention group and decreased in the control group. However, no difference between the two study groups was found based on GEE analysis." "The challenge with low usage of behavioral change services has been revealed also in earlier studies [48]." "...there were no change in anthropometrics or fitness in the

22-ii) Highlight unanswered new questions, suggest future research

Highlight unanswered new questions, suggest future research.

	1	2	3	4	5	
subitem not at all important	○	○	○	○	○	essential

Does your paper address subitem 22-ii?

Copy and paste relevant sections from the manuscript (include quotes in quotation marks "like this" to indicate direct quotes from your manuscript), or elaborate on this item by providing additional information not in the ms, or briefly explain why the item is not applicable/relevant for your study

"However, in future studies more attention must be paid to the continuation of the use of a service. Other target populations need also be investigated in future."

20) Trial limitations, addressing sources of potential bias, imprecision, and, if relevant, multiplicity of analyses

20-i) Typical limitations in ehealth trials

Typical limitations in ehealth trials: Participants in ehealth trials are rarely blinded. Ehealth trials often look at a multiplicity of outcomes, increasing risk for a Type I error. Discuss biases due to non-use of the intervention/usability issues, biases through informed consent procedures, unexpected events.

	1	2	3	4	5	
subitem not at all important	○	○	○	○	○	essential

Does your paper address subitem 20-i? *

Copy and paste relevant sections from the manuscript (include quotes in quotation marks "like this" to indicate direct quotes from your manuscript), or elaborate on this item by providing additional information not in the ms, or briefly explain why the item is not applicable/relevant for your study

"One limitation of this study was the missing PA data, the amount of which increased toward the end of the trial." "Another limitation was the narrow age range which limits the generalization of the results. Any long-term follow-up PA measurement after the end of the 6-month trial was not conducted in this study which can also be seen as a limitation."

21) Generalisability (external validity, applicability) of the trial findings

NPT: External validity of the trial findings according to the intervention, comparators, patients, and care providers or centers involved in the trial

21-i) Generalizability to other populations

Generalizability to other populations: In particular, discuss generalizability to a general Internet population, outside of a RCT setting, and general patient population, including applicability of the study results for other organizations

	1	2	3	4	5	
subitem not at all important	○	○	○	○	○	essential

Does your paper address subitem 21-i?

Copy and paste relevant sections from the manuscript (include quotes in quotation marks "like this" to indicate direct quotes from your manuscript), or elaborate on this item by providing additional information not in the ms, or briefly explain why the item is not applicable/relevant for your study

"Another limitation was the narrow age range which limits the generalization of the results." "Other target populations need also be investigated in future."

21-ii) Discuss if there were elements in the RCT that would be different in a routine application setting

Discuss if there were elements in the RCT that would be different in a routine application setting (e.g., prompts/reminders, more human involvement, training sessions or other co-interventions) and what impact the omission of these elements could have on use, adoption, or outcomes if the intervention is applied outside of a RCT setting.

	1	2	3	4	5	
subitem not at all important	○	○	○	○	○	essential

Does your paper address subitem 21-ii?

Copy and paste relevant sections from the manuscript (include quotes in quotation marks "like this" to indicate direct quotes from your manuscript), or elaborate on this item by providing additional

information not in the ms, or briefly explain why the item is not applicable/relevant for your study

Not applicable in this study.

OTHER INFORMATION

23) Registration number and name of trial registry

Does your paper address CONSORT subitem 23? *

Copy and paste relevant sections from the manuscript (include quotes in quotation marks "like this" to indicate direct quotes from your manuscript), or elaborate on this item by providing additional information not in the ms, or briefly explain why the item is not applicable/relevant for your study

"the clinical trials register NCT01376986"

24) Where the full trial protocol can be accessed, if available

Does your paper address CONSORT subitem 24? *

Cite a Multimedia Appendix, other reference, or copy and paste relevant sections from the manuscript (include quotes in quotation marks "like this" to indicate direct quotes from your manuscript), or elaborate on this item by providing additional information not in the ms, or briefly explain why the item is not applicable/relevant for your study

See the protocol paper - Ahola et al. (2013) Gamified physical activation of young men – a Multidisciplinary Population-Based Randomized Controlled Trial (MOPO study).

25) Sources of funding and other support (such as supply of drugs), role of funders

Does your paper address CONSORT subitem 25? *

Copy and paste relevant sections from the manuscript (include quotes in quotation marks "like this" to indicate direct quotes from your manuscript), or elaborate on this item by providing additional information not in the ms, or briefly explain why the item is not applicable/relevant for your study

This study was funded by the Ministry of Education and Culture, Centre for Military Medicine, Centre for Economic Development, Transport and the Environment of North Ostrobothnia, the Finnish Funding Agency for Technology and Innovation, the Northern Ostrobothnia Hospital District, Infotech Oulu, and the Finnish Cultural Foundation. The funders had no role in the planning or implementation of the intervention.

X27) Conflicts of Interest (not a CONSORT item)

X27-i) State the relation of the study team towards the system being evaluated

In addition to the usual declaration of interests (financial or otherwise), also state the relation of the study team towards the system being evaluated, i.e., state if the authors/evaluators are distinct from or identical with the developers/sponsors of the intervention.

	1	2	3	4	5	
subitem not at all important	○	○	○	○	○	essential

Does your paper address subitem X27-i?

Copy and paste relevant sections from the manuscript (include quotes in quotation marks "like this" to indicate direct quotes from your manuscript), or elaborate on this item by providing additional information not in the ms, or briefly explain why the item is not applicable/relevant for your study

"Riikka Ahola is currently an employee of Polar Electro. All other authors of this paper declare no conflicts of interest."

About the CONSORT EHEALTH checklist

As a result of using this checklist, did you make changes in your manuscript? *

- ○ yes, major changes
- ◉ yes, minor changes
- ○ no

What were the most important changes you made as a result of using this checklist?

How much time did you spend on going through the checklist INCLUDING making changes in your manuscript *

15h

As a result of using this checklist, do you think your manuscript has improved? *

- (x) yes
- ( ) no
- ( ) Other:

Would you like to become involved in the CONSORT EHEALTH group?

This would involve for example becoming involved in participating in a workshop and writing an "Explanation and Elaboration" document

- ( ) yes
- ( ) no
- ( ) Other:

Any other comments or questions on CONSORT EHEALTH

The limited word count of the form challenged the direct copying of the text from the manuscript. That is one reason why the fulfillment of the form took so much time.

STOP - Save this form as PDF before you click submit

To generate a record that you filled in this form, we recommend to generate a PDF of this page (on a Mac, simply select "print" and then select "print as PDF") before you submit it.

When you submit your (revised) paper to JMIR, please upload the PDF as supplementary file.

Don't worry if some text in the textboxes is cut off, as we still have the complete information in our database. Thank you!

Final step: Click submit !

Click submit so we have your answers in our database!

Submit

Never submit passwords through Google Forms.

Powered by

This content is neither created nor endorsed by Google.

Report Abuse - Terms of Service - Additional Terms
